# Infection of Adult Thymus with Murine Retrovirus Induces Virus-Specific Central Tolerance That Prevents Functional Memory CD8^+^ T Cell Differentiation

**DOI:** 10.1371/journal.ppat.1003937

**Published:** 2014-03-20

**Authors:** Shiki Takamura, Eiji Kajiwara, Sachiyo Tsuji-Kawahara, Tomoko Masumoto, Makoto Fujisawa, Maiko Kato, Tomomi Chikaishi, Yuri Kawasaki, Saori Kinoshita, Manami Itoi, Nobuo Sakaguchi, Masaaki Miyazawa

**Affiliations:** 1 Department of Immunology, Kinki University Faculty of Medicine, Osaka, Japan; 2 Department of Immunology and Microbiology, Meiji University of Integrative Medicine, Kyoto, Japan; 3 Department of Immunology, Kumamoto University School of Medicine, Kumamoto, Japan; Washington University School of Medicine, United States of America

## Abstract

In chronic viral infections, persistent antigen presentation causes progressive exhaustion of virus-specific CD8^+^ T cells. It has become clear, however, that virus-specific naïve CD8^+^ T cells newly generated from the thymus can be primed with persisting antigens. In the setting of low antigen density and resolved inflammation, newly primed CD8^+^ T cells are preferentially recruited into the functional memory pool. Thus, continual recruitment of naïve CD8^+^ T cells from the thymus is important for preserving the population of functional memory CD8^+^ T cells in chronically infected animals. Friend virus (FV) is the pathogenic murine retrovirus that establishes chronic infection in adult mice, which is bolstered by the profound exhaustion of virus-specific CD8^+^ T cells induced during the early phase of infection. Here we show an additional evasion strategy in which FV disseminates efficiently into the thymus, ultimately leading to clonal deletion of thymocytes that are reactive to FV antigens. Owing to the resultant lack of virus-specific recent thymic emigrants, along with the above exhaustion of antigen-experienced peripheral CD8^+^ T cells, mice chronically infected with FV fail to establish a functional virus-specific CD8^+^ T cell pool, and are highly susceptible to challenge with tumor cells expressing FV-encoded antigen. However, FV-specific naïve CD8^+^ T cells generated in uninfected mice can be primed and differentiate into functional memory CD8^+^ T cells upon their transfer into chronically infected animals. These findings indicate that virus-induced central tolerance that develops during the chronic phase of infection accelerates the accumulation of dysfunctional memory CD8^+^ T cells.

## Author Summary

During thymocyte development, cells that recognize self-antigens are specifically deleted by the process known as negative selection. However, some pathogens disseminate to the thymus, and can induce foreign antigen presentation within this organ, resulting in potentially harmful clonal deletion of pathogen-specific T-lymphocyte precursors. In chronic infections, pathogen-specific T cells in the periphery progressively lose their functionality due to continual stimulation with the persisting antigen, a phenomenon known as T cell exhaustion. However, pathogen-reactive naïve T cells freshly primed during the chronic phase of infection can nevertheless replenish the functional pool of memory T cells. Therefore, a lack of their generation in the face of peripheral exhaustion may ultimately cause the loss of functional memory T cells and the resultant lack of pathogen control. In this study, we demonstrate that Friend murine retrovirus can utilize the above immune evasion strategy, a combination of ongoing peripheral exhaustion and virus-induced central tolerance. Our data suggest that, along with the reinvigoration of exhausted T cells in the periphery, preservation of the thymic function in supplying pathogen-specific naïve T cells may be important when considering immunological control of chronic infection with thymotropic pathogens.

## Introduction

Antigen-specific CD8^+^ T cell populations are a major component that eliminate cells infected with intracellular pathogens. After infections that are cleared acutely, antigen-specific CD8^+^ T cells can differentiate into functionally competent memory CD8^+^ T cells, and can persist for a long time in the apparent absence of relevant antigens [1]. In contrast, in the case of chronic infections where the antigens are presented persistently, CD8^+^ T cells primed during the early phase of infection succumb to progressive functional defects, such as impaired ability to proliferate, kill infected cells, and/or produce effector cytokines in response to the antigen-specific stimulation [2]. In most cases, this loss of effector functions is due to signaling through inhibitory molecules such as programmed cell death 1 (PD-1), lymphocyte activation gene 3 (LAG-3), CD244, CD160, and T cell Ig domain and mucin domain 3 (Tim-3), and is called exhaustion [2]. The severity of this dysfunction, which is in correlation with the numbers and extent of inhibitory molecules expressed on exhausted CD8^+^ T cells, is critically linked with the levels of repetitive exposure to the relevant antigen [3]. In addition to their negative effects on the functionality of antigen-experienced CD8^+^ T cells, persisting antigens also induce stable proliferation of already-exhausted memory CD8^+^ T cells [4]. The resultantly sustained numbers of functionally impaired memory CD8^+^ T cells potentially inhibit optimal priming of otherwise functional fresh memory CD8^+^ T cells via physiological competition for the niche. Thus, chronic infection is a vicious circle of ongoing CD8^+^ T cell dysfunction and ineffective antigen clearance. Despite such detrimental effects, however, recent studies shed light on a beneficial role of persistent antigens on the functionalities of memory CD8^+^ T cells. Naïve CD8^+^ T cells are continuously provided from the thymus even during the chronic phase of infection, and this continual thymic output can result in the priming of new antigen-specific CD8^+^ T cells [5]. Unlike exhausted CD8^+^ T cells that were primed in the early phase of infection, CD8^+^ T cells primed during the chronic phase of infection in low-antigen and less intensive inflammatory settings give rise to functional memory CD8^+^ T cells capable of mounting authentic recall responses [6]. Similar memory-dominated differentiation of CD8^+^ T cells can also be found when CD8^+^ T cells are primed after the peak of an acute infection, by the time that the majority of antigens have been cleared out [7]. Thus, persistent antigens play an important role in generating functional memory CD8^+^ T cells in the presence of continual thymic output.

Friend virus (FV) is a murine retrovirus complex comprising two gammaretroviruses: replication-competent Friend murine leukemia virus (F-MuLV) and replication-defective spleen focus-forming virus (SFFV) [8]. Although FV particles can bind onto the surfaces of a wide variety of cells via cationic amino acid transporter as an entry receptor [9], [10], viral replication occurs preferentially in actively dividing hematopoietic cells, especially in erythroid progenitor cells in the bone marrow (BM) and spleen [8]. In the susceptible strains of mice [e.g. (C57BL/6×A/WySnJ)F_1_ (B6AF_1_) mice], SFFV gp55 envelope glycoprotein stimulates erythropoietin receptor in conjunction with its binding to the short form of hematopoietic cell-specific receptor tyrosine kinase Stk/Ron, resulting in massive expansion of virus-infected erythroblasts [11], [12]. As a result, FV-infected mice develop severe splenomegaly associated with polycythemia and high-level viremia [8]. This acute pathogenesis ultimately causes leukemia development in some cases, but most B6AF_1_ mice recover from the above initial splenomegaly and survive. Following recovery, however, FV establishes a lifelong chronic infection and mice are never able to completely eradicate this virus [13]. Although antigen levels are low during the chronic phase of infection, it is sufficient to prime antigen-specific CD8^+^ T cells, as adoptively transferred FV-specific CD8^+^ T cells can be activated and acquire effector functions in chronically infected animals [14]. However, the precise effect of persistent antigens on the functionalities of FV-specific memory CD8^+^ T cells remains unclear.

It has been proposed that FV-specific CD8^+^ T cells progressively lose their effector functions during the chronic phase of infection due to suppression by virus-induced regulatory T cells (Tregs) [14]–[20]. In B6AF_1_ mice, however, we found that severe dysfunction of FV-specific CD8^+^ T cells appeared even during the acute phase of infection in the absence of an apparent increase in Treg functions, and this was critically related with the FV-induced acute pathogenesis [21]. At the peak of infection, numbers of virus-infected erythroblasts in the spleen reached more than 10^8^, comprising approximately 75% of total splenocytes. These virus-infected erythroblasts intensively expressed programmed death ligand 1 (PD-L1) and MHC class I, thereby creating a highly tolerogenic environment. Consequently, FV-specific effector CD8^+^ T cells suffered rapid exhaustion, and most of these cells were incapable of responding to restimulation with FV-encoded antigens [21]. Since effector CD8^+^ T cells generated in the absence of the splenomegaly in mice inoculated with F-MuLV alone showed less exhausted phenotypes as compared to those generated in the presence of splenomegaly, SFFV-induced pathogenesis was shown to be mainly responsible for the severe and rapid exhaustion of antigen-specific effector CD8^+^ T cells [21]. In the current study, we further extended our previous study, and found that functional memory CD8^+^ T cells were rarely detected in FV-infected mice even during the chronic phase of infection. This severe dysfunction was apparently exclusive to FV-specific CD8^+^ T cells but not found in FV-unrelated CD8^+^ T cells. As a basis for this antigen-specific memory T cell dysfunction, FV is shown to disseminate to the thymus and induce virus-specific central tolerance, thereby preventing the generation of virus-specific naïve CD8^+^ T cells. Thus, the severe dysfunction of FV-specific memory CD8^+^ T cells is likely due to a combination of ongoing peripheral exhaustion and the deletion of virus-reactive thymocytes.

## Results

### FV disseminates to and persists in the thymus following infection

Infection of B6AF_1_ mice with FV results in the expansion of virus-infected erythroblasts that causes severe exhaustion of antigen-specific CD8^+^ T cells even during the acute phase of infection [21]. During the chronic phase of FV infection where the progressive expansion of virus-infected erythroblasts has resolved, FV-specific naïve CD8^+^ T cells must be continuously provided from the thymus. If this is the case, newly recruited FV-specific naïve CD8^+^ T cells should be primed with persistent FV antigens in the absence of expanded virus-infected erythroblasts. These newly primed CD8^+^ T cells may then participate in the FV-specific CD8^+^ T cell responses with better functionalities than the above exhausted cells [5]. However, most of the virus-specific memory CD8^+^ T cells in the BM and spleen still retained highly exhausted phenotypes even more than 6 weeks after infection, as determined by the expression of multiple inhibitory receptors, PD-1, LAG-3, and Tim-3 on their surfaces (Figure S1A, B). Levels of expression of these inhibitory receptors were much higher on virus-specific CD8^+^ T cells in the BM than on those in the spleen, indicating that during the chronic phase of infection, memory CD8^+^ T cells received more abundant antigenic stimulation in the former tissue (Figure S1B). As most virus-specific CD8^+^ T cells succumb to irreversible exhaustion even during the early phase of infection [21], the above phenotypically exhausted memory CD8^+^ T cells in chronically infected mice were no longer reinvigorated by the administration of PD-1-blocking antibody (data not shown). In support of this, almost no FV antigen-reactive IFN-γ production from CD8^+^ T cells was detected when FV-infected mice were later challenged with FBL3 tumor cells, a potent inducer of FV-specific CD8^+^ T cell responses, and the cells were restimulated with the MHC class I-restricted viral antigenic peptide (Figure S1C, D). These virus-infected mice also failed to control FBL3 tumor progression, similar to those that received syngenic tumor cells, EL-4, while uninfected mice rapidly rejected the tumor (Figure S1E, F). Based on these observations, we started to ask if FV infection influences the continuous recruitment of virus-specific naïve CD8^+^ T cells from the thymus.

**Figure 1:**
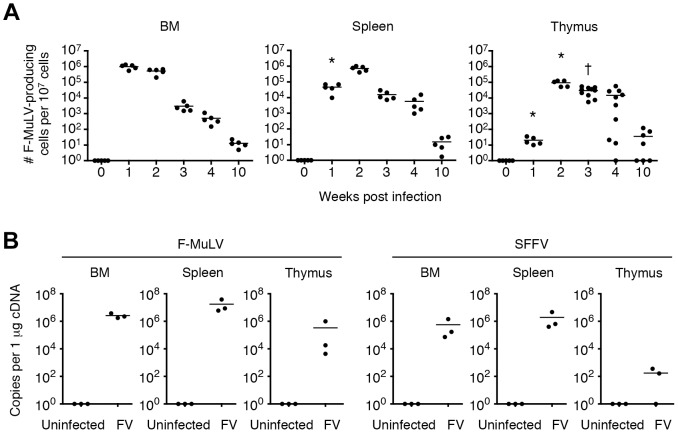
FV disseminates to and persists in the thymus following infection. Mice were infected with 1,000 SFFU of FV. (A) Cells in the BM, spleen and thymus were isolated at indicated time-points, and were cocultured with *M. dunni* cells to enumerate F-MuLV infectious centers. Each symbol represents an individual mouse. Data are representative of two independent experiments with essentially equivalent results. *, *p*<0.0001 in comparison with the numbers of FV-producing cells in the BM at the same time-point; †, *p*<0.0001 in comparison with those in the spleen, by two-way ANOVA with Bonferroni's correction for multiple comparisons. (B) Total RNA was purified from the BM, spleen and thymus of FV-infected mice at day 14. Expression levels of F-MuLV and SFFV mRNA were analyzed by quantitative real-time PCR assays. Shown are copy numbers of viral DNA fragments amplified from 1 µg of total cDNA.

**Figure 2:**
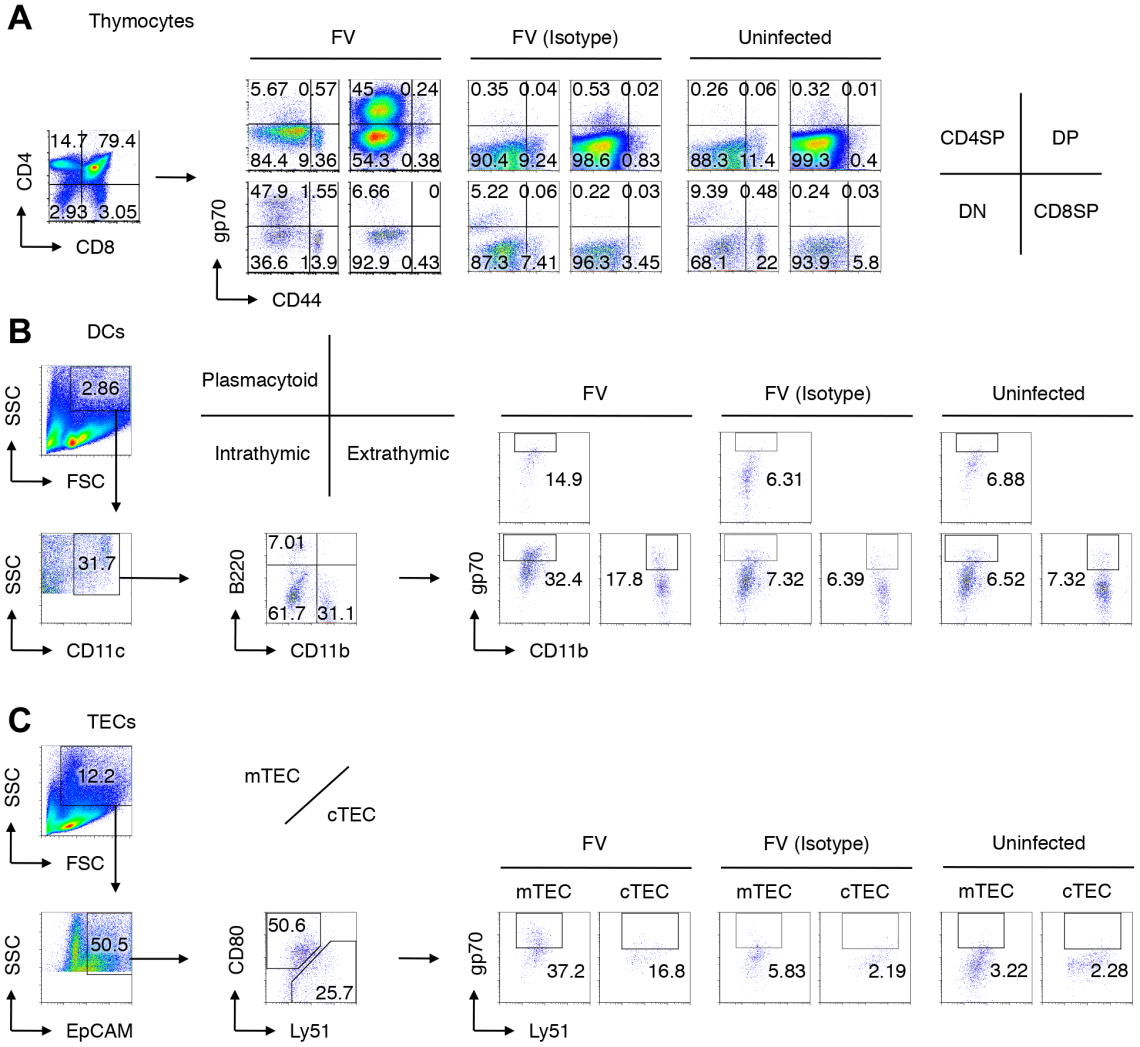
Viral antigen expression in each cell population in the thymus after FV infection. Mice were infected with 1,000 SFFU of FV. At day 14 after infection, cells in the thymus were isolated and stained with indicated Abs. Shown are representative staining patterns and gating protocols of thymocytes (A), thymic DCs (B), and TEC populations (C). (B) Cells purified from the thymus were incubated with microbeads-labeled anti-CD90.2 antibody, and antibody-negative populations were further stained with fluorescent-labeled anti-CD11b, anti-CD11c, anti-B220, and anti-gp70. CD11c^+^ cells were separated into B220^+^ plasmacytoid DCs, B220^−^CD11b^−^ DCs of intrathymic origin, and CD11b^+^ DCs of extrathymic origin. (C) CD90.2^−^ populations were stained with fluorescent-labeled anti-EpCAM, anti-CD80, anti-Ly51, and anti-gp70. EpCAM^+^ cells were separated into CD80^+^ medullary TECs (mTECs), and Ly51^+^ cortical TECs (cTECs). Nonspecific binding of the biotinylated anti-gp70 mAb 720 especially onto DN thymocytes, DCs and TECs was inevitable even in the presence of anti-Fc receptor Abs, as evidenced by the background staining with the isotype control IgG. Thus, the percentages of “gp70^+^” cells shown here include some background values.

**Figure 3:**
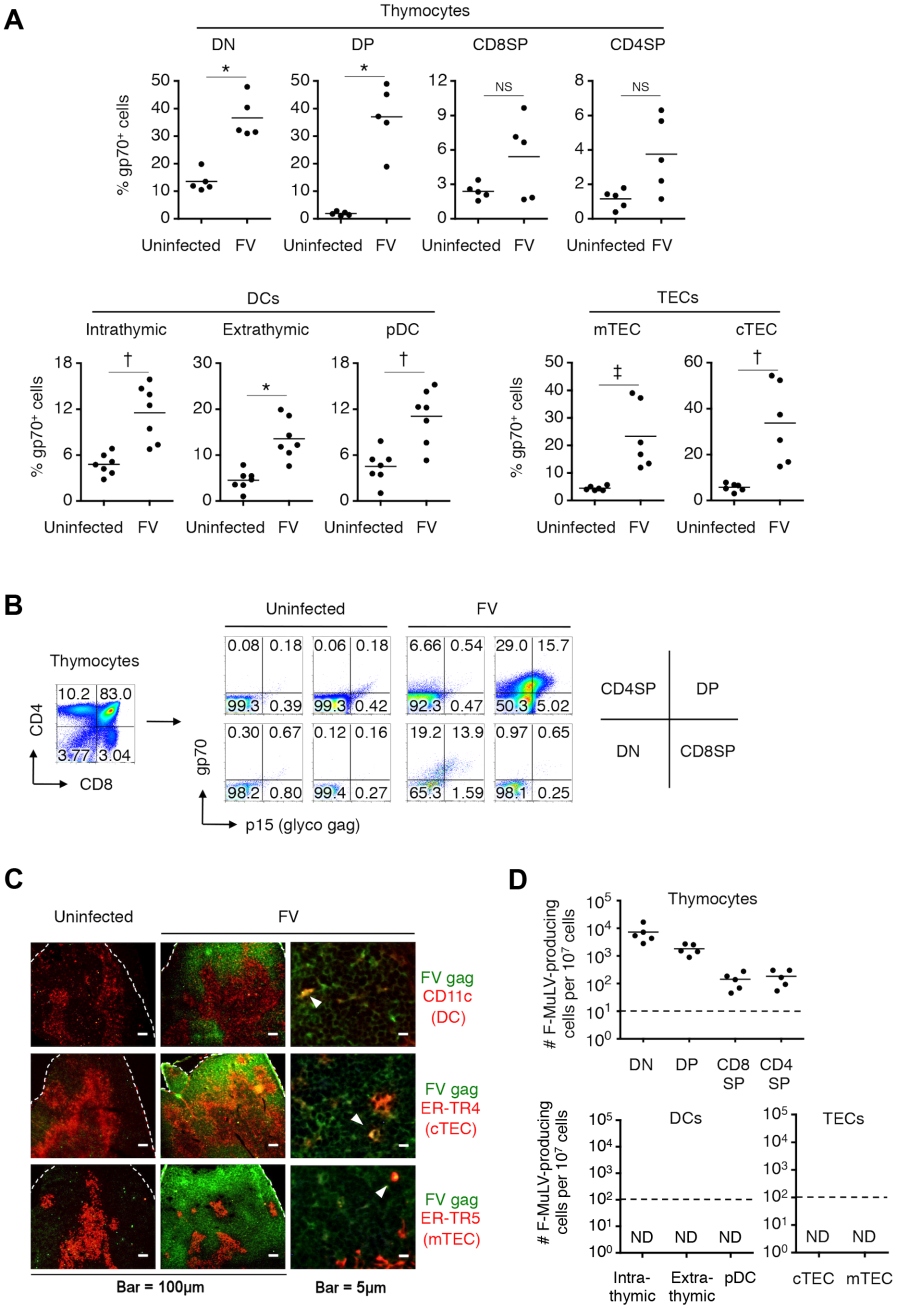
Identification of FV-infected cells in the thymus. Mice were infected with 1,000 SFFU of FV. (A) At day 14 post infection, cells in the thymus were isolated and stained with the indicated Abs. Shown are frequencies of F-MuLV gp70^+^ cells among indicated populations as described in Figure 2. Differences in means between uninfected and FV-infected groups were analyzed by two-way ANOVA with Bonferroni's corrections for multiple comparisons: *, *p*<0.0001; †, *p*<0.001; ‡, *p*<0.05. (B) Shown are representative staining patterns for cell surface gp70 and p15*^gag^* of each thymocyte population. (C) Representative frozen sections of the thymus from FV-infected mice (14 days post infection) were stained for CD11c and F-MuLV gag p30 (top), cTEC-specific ER-TR4 and F-MuLV gag p30 (middle), or mTEC-specific ER-TR5 and F-MuLV gag p30 (bottom). Arrowheads indicate cells double positive for the indicated cell surface marker and the viral antigen. A larger view field of the sections shown here can be seen in Figure S3. (D) Mice were infected with 1,000 SFFU of FV. At day 14 after infection, cells in the thymus were isolated, stained with the indicated Abs, and FACS sorted into the indicated populations as described in Figure 2. Cells were cocultured with *M. dunni* cells to enumerate F-MuLV infectious centers. Each symbol represents cells from an individual mouse. Dashed lines indicate the detectable limits (e.g. maximum available numbers of DCs and TECs used in this experiment were 1×10^5^). Data are representative of two independent experiments with essentially equivalent results.

It has been reported that the BM and spleen are the major organs where massive replication of FV takes place [8]. Large numbers of virus-producing cells were already found in the BM at as early as 1 week post infection, and their numbers in the spleen reached a peak by 2 weeks post infection (Figure 1A). Despite a gradual decline after the peak of infection, significant numbers of virus-producing cells could still be detected until at least 10 weeks post infection, indicating that FV established persistent infection in these organs (Figure 1A). Surprisingly, significant numbers of virus-producing cells were also detected in the thymus at 2 weeks post infection, followed by the establishment of persistent infection in this organ (Figure 1A). At 2 weeks post FV infection, both F-MuLV and SFFV mRNA were detected from the thymus, but the levels of SFFV mRNA expression were much lower than those of F-MuLV mRNA in this organ (Figure 1B). Using the monoclonal antibody specific for F-MuLV envelope glycoprotein gp70 we found that it is mainly double positive (DP) and double negative (DN) thymocytes that harbor F-MuLV in the thymus, as up to 50% of cells in these populations were expressing gp70 on their surfaces at the peak of infection (Figure 2A and Figure 3A). Surface expression of F-MuLV gag p15 antigen was also detected on gp70-expressing DN and DP thymocytes (Figure 3B), indicating the presence of glycosylated gag protein (gPr75*^gag^*). Further, when thymocytes were fixed and permeabilized, F-MuLV capsid p30 antigen was also detected in gp70^+^ DN and DP thymocytes (data not shown). These results indicate that F-MuLV gene products were actively synthesized in DN and DP thymocytes. Immunohistochemical analyses also revealed viral gag antigen expression throughout the cortex where the DN and DP thymocytes were distributed (Figure 3C). Interestingly, the spread of the virus into the thymus appeared to be delayed a week as compared to that in the BM and spleen (Figure 1), suggesting that the dissemination of the virus to the thymus occurred following massive viral replication in the BM and spleen. In support of this, only marginal increases in proportions of gp70^+^ cells in the thymus were observed when *Fv2^r^* C57BL/6 (B6) mice lacking the expression of the short form of STK (sf-Stk) were infected with FV or B6AF_1_ mice were inoculated with F-MuLV alone (Figure S2). Thus SFFV-induced proliferation of FV-infected erythroid cells is required for effective dissemination of FV into the thymus.

Notably, CD44^hi^ DN thymocytes virtually lacked the expression of gp70, indicating that T cell progenitors migrated from the BM were not an initial source of virus dissemination to the thymus (Figure 2A). Since FV proviruses are known to be preferentially integrated into proliferating cells, and a large proportion of CD44^−^ DN thymocytes were gp70^+^, it is likely that FV initially infects DN thymocytes within this organ, and then replicates vigorously in DN thymocytes that proliferate and differentiate into DP thymocytes. The highest number of virus-producing cells in the DN population among thymocytes also supports this idea (Figure 3D). The expression of FV antigens was also found on the surfaces of all thymic DC populations as well as on medullary (mTECs) and cortical thymic epithelial cells (cTECs) (Figure 2B, C, Figure 3A, C, and Figure S3). Unlike the virus-infected thymocytes, however, the production of infectious virus particles was not detected from the thymic DC and TEC populations, at least by infectious center assays (Figure 3D). These results suggest that although cell-bound viral antigens are detectable in most cell types in the thymus, productive viral replication occurs preferentially in the thymocytes.

### Infection of the thymus with FV leads to clonal deletion of FV-specific thymocytes

**Figure 4:**
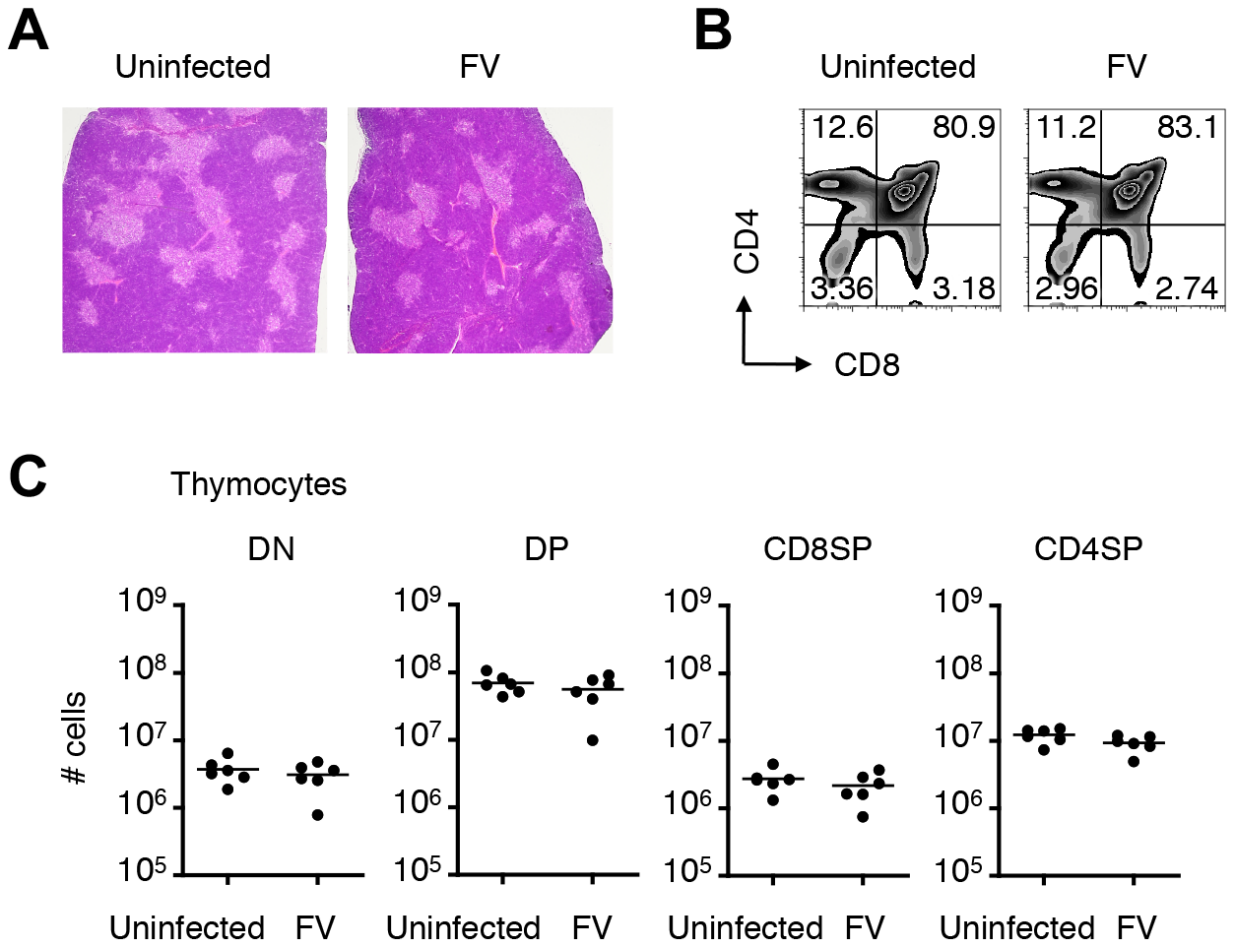
Infection of thymus with FV has no influence on numbers and frequencies of thymocyte populations. Mice were infected with 1,000 SFFU of FV. (A) Representative hematoxylin- and eosin-stained thymus sections from FV-infected (21 days post infection) or age-matched uninfected mice. Thymocytes isolated from the same experimental mice were stained for CD4 and CD8. Shown are representative dot plots (B) and actual numbers of each thymocyte population (C). Each symbol represents an individual mouse. No significant differences were observed between the groups. Data are representative of two independent experiments with essentially equivalent results.

Given the global nature of FV infection in the thymus, we anticipated that the general function of the thymus in FV-infected mice might be affected. Unlike the spleen, where the physiological architecture was significantly disrupted by the massive expansion of erythroblasts, the vigorous viral replication had no impact on the size of the thymus (data not shown), and caused no apparent morphologic abnormality even at the peak of infection (Figure 4A). The frequencies and absolute numbers of each thymocyte population in FV-infected mice were also not different from those in age-matched uninfected mice (Figure 4B, C). These results so far revealed that the basic function of the thymus appeared to be retained in FV-infected mice.

**Figure 5:**
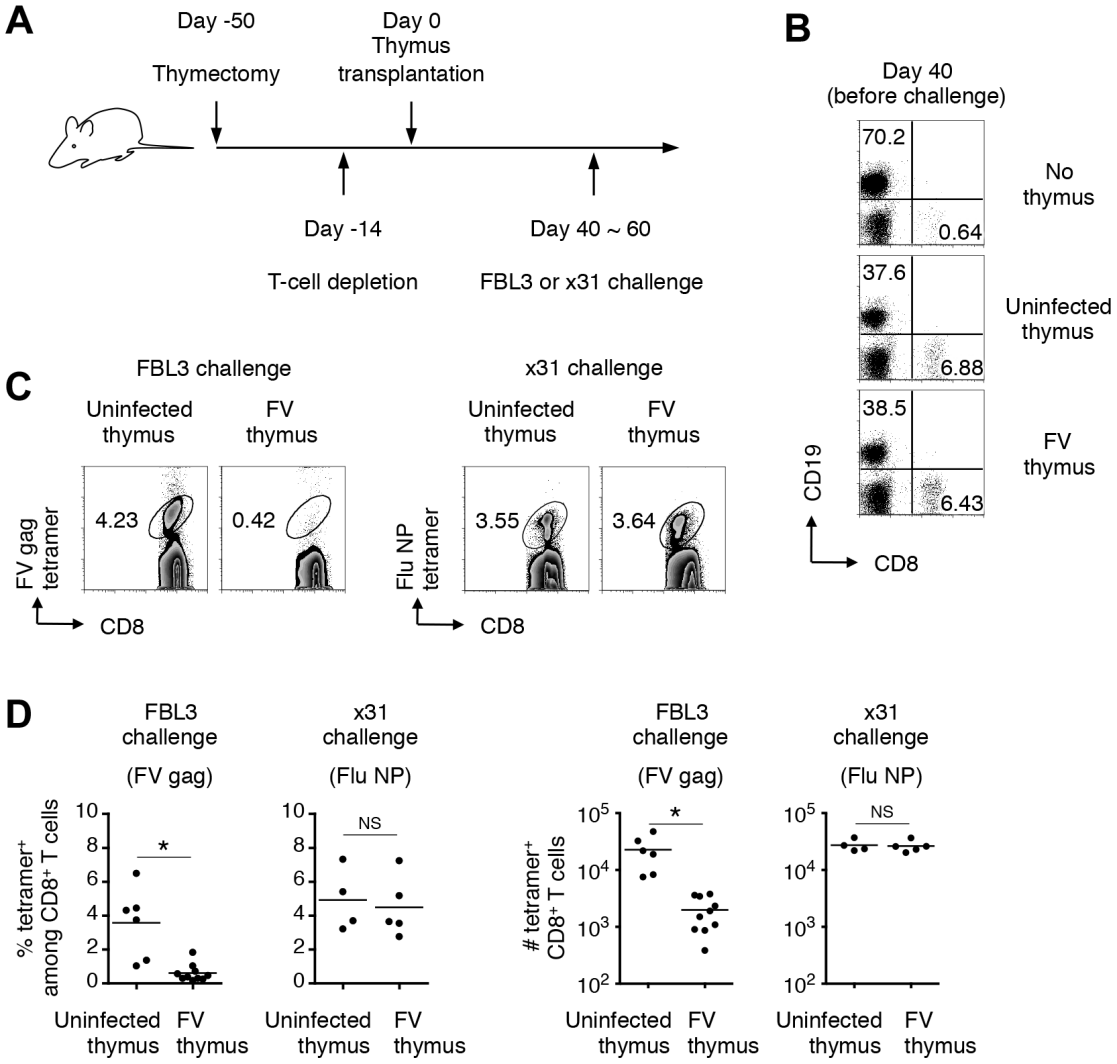
Infection of thymus with FV leads to clonal deletion of FV-specific thymocytes. (A) To make a T cell-free microenvironment in B6AF_1_ mice, day 5 neonatal pups were thymectomized, and 5 weeks later were injected intraperitoneally with depleting Abs for both CD4 and CD8. Two weeks later, a thymic lobe from either FV-infected (2–3 weeks post infection) or age-matched uninfected mice was grafted under the kidney capsule. (B) At day 40 after transplantation, splenocytes were isolated and stained with the indicated Abs. Shown are representative staining patterns for CD8 and CD19. (C) Six weeks after transplantation, when peripheral CD8 T cells were reconstituted, mice were challenged with either FV-antigen bearing FBL3 tumor cells or influenza virus ×31. Splenocytes were isolated at 10–14 days post challenge, and stained with the indicated Abs and either F-MuLV gag_75–83_/D^b^ or Flu NP_366–374_/D^b^ tetramer. Shown are representative staining patterns for CD8 and each tetramer among CD8^+^ T cells. (D) Shown are frequencies of tetramer^+^ cells among CD8^+^ T cells (left panels), and actual numbers of tetramer^+^ CD8^+^ T cells (right panels). Averages of % and actual numbers of tetramer^+^ cells were compared between uninfected and FV-infected groups by two-tailed Welch's *t*-test, as variances in both cases were not regarded as equal. *, *p*<0.021; NS, not significant.

However, since viral antigen expression was found in the thymic DCs and TECs (Figure 2 and 3), we speculated that processed viral antigens could be presented in the context of MHC class I and class II molecules on the surfaces of these cells, which might eliminate thymocytes bearing viral antigen-reactive T-cell receptors (TCRs) via negative selection. To test this, we employed a thymus transplantation approach in which thymic lobes from either FV-infected (4–8 weeks post infection) or age-matched uninfected mice were transplanted into syngenic recipients that have been thymectomized and treated with T-cell-depleting antibodies prior to the transplantation (Figure 5A). Transplantation of thymic lobes from FV-infected or uninfected mice was equally able to reconstitute peripheral T cells as we observed similar frequencies of CD8^+^ T cells in the blood at 6–8 weeks post transplantation (Figure 5B). Note that we have checked spleen weights and F-MuLV gp70 expression on B cells and Ter119^+^ erythroblasts in the recipient mice that received the thymus from FV-infected donors, and found no evidence of splenomegaly or significant increase in gp70^+^ cells (Figure S4). These results clearly indicate minimal, if any, transmission of FV via the transplantation of thymic lobes from FV-infected donors. In both recipient groups, comparable frequencies and numbers of influenza nucleoprotein (NP)-specific CD8^+^ T cells were detected in the spleens following infection with influenza virus ×31 (Figure 5C, D), suggesting that CD8^+^ T cells that arose from the FV-infected thymic transplant were bona fide mature T lymphocytes with the intact capability to respond to an FV-unrelated antigen. Importantly, however, following challenge with FV antigen-bearing FBL3 tumor cells, minimal evidence of FV-specific tetramer binding on CD8^+^ T cells was observed in mice transplanted with FV-infected thymuses, while control mice transplanted with uninfected thymuses showed significantly higher levels of FV-specific CD8^+^ T cell expansion at the peak of response (Figure 5C, D). This was indicative of an incapability of FV-infected thymus to generate FV-specific naïve CD8^+^ T cells. Overall, the above data clearly demonstrated that thymic infection with FV resulted in the depletion of thymocytes expressing TCRs specific for FV antigens.

### Thymic DCs and TECs are the direct deleters of FV-specific thymocytes

**Figure 6:**
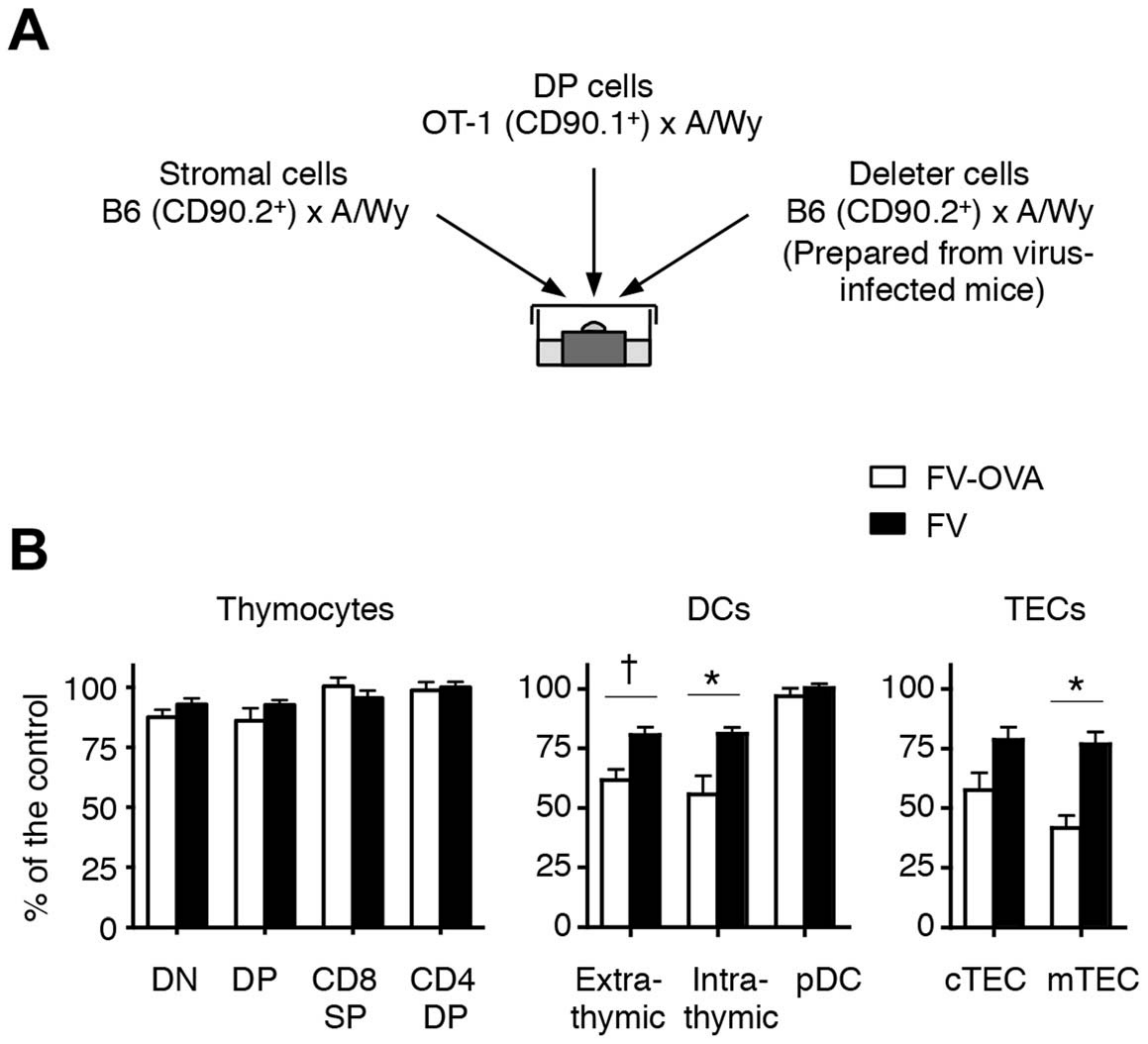
Thymic DCs and TECs are the major deleters of FV-specific thymocytes. (A) Thymic stromal cells of B6AF_1_ mice were prepared from E15.5 fetal thymus lobes. OT-1 DP thymocytes were sorted from adult (OT-1-Thy1.1× A/WySnJ)F_1_ mice. Thymocytes, thymic DCs and TECs from FV-OVA infected mice (21 days post infection) were sorted into each population as indicated in Figure 2. Cells were mixed and cultured as a reassembled organ for 4 days. (B) Shown are relative percentages of CD8 SP cells as compared to the control. The percentage of the control value was calculated by [(% of CD8 SP cells in the experimental culture)/(% of CD8 SP cells in the control culture)]×100 (see Figure S5). Averages were compared between FV-OVA- and FV-infected groups for each third cell population by two-way ANOVA with Bonferroni's correction for multiple comparisons, and statistically significant differentiations are indicated: *, *p*<0.01; †, *p*<0.05.

Given that the dissemination of FV to the thymus led to the apparent negative selection of viral antigen-reactive thymocytes, we next wished to determine which cell populations in the FV-infected thymus were responsible for this negative selection. To do this, we performed the fetal thymus organ culture (FTOC) [22]. To utilize TCR-transgenic T cells, we first generated a recombinant F-MuLV, F-MuLV-OVA, expressing the K^b^-restricted OVA-derived peptide OVA_257–264_, for which the OT-1 CD8^+^ T cells are reactive, and the activation of OT-1 CD8^+^ T cells upon stimulation with F-MuLV-OVA-infected cells was confirmed (Figure S5). As the SFFV-induced massive proliferation of erythroid cells was required for effective dissemination of F-MuLV into the thymus (Figure S2), mice were either inoculated with FV alone (a mixture of approximately equal infectious titers of F-MuLV and SFFV) or FV plus F-MuLV-OVA (abbreviated as FV-OVA). To ensure that F-MuLV-OVA could infect SFFV-infected erythroid cells by overcoming the possible receptor interference with wild-type F-MuLV, a twice larger infectious titer of F-MuLV-OVA was given relative to the titer of F-MuLV in the FV complex used. A mixture of thymocyte-depleted thymic stromal cells from uninfected fetus (day 15 of gestation) and FACS-sorted OT-1 DP thymocytes were cultured in the presence of a third population (each single population purified from the thymus of either FV- or FV-OVA-infected mice) as a candidate deleter cell population (Figure 6A). In the absence of the third population, the development of OT-1 CD8 single positive (SP) cells was observed at day 5, which was apparently reduced when OVA-expressing E.G7 cells were added in the culture (Figure S6A). The addition of control EL-4 cells did not affect the generation of CD8 SP cells, revealing the reliability of this approach to test the ability of the third population to induce antigen-specific negative selection. Because the numbers of OT-1 CD8 SP cells recovered from the FTOC varied significantly in each experiment, perhaps due to the technical difficulty in simultaneously preparing three different groups of cells (stromal cells, OT-1 DP thymocytes, and third populations purified from the thymus of virus-infected mice; see Figure 6A), we evaluated the influences of third populations on the development of OT-1 CD8 SP cells by calculating the relative reduction of the frequency of OT-1 CD8 SP cells in an experimental culture compared to those in the control culture (without adding the third population) in each set of experiments (Figure S6B). As shown in Figure 6B, the addition of thymocyte populations from FV-OVA-infected mice had no influence on the development of OT-1 CD8 SP cells, indicating that despite the high level of FV infection and extensive antigen expression, FV-infected thymocytes did not actively induce the negative selection of viral antigen-specific thymocytes. In contrast, we observed a significant reduction in the frequencies of OT-1 CD8 SP cells when the cultures contained either thymic DC populations (except pDC) or TEC populations from FV-OVA-infected mice (Figure 6B and Figure S6B). Although some reductions in the frequency of OT-1 CD8 SP cells were also observed when DCs and TECs from FV-infected mice were added in the culture, the extent of CD8 SP cell reduction was significantly higher when thymic DCs or mTEC from FV-OVA-infected mice were added (Figure 6B and Figure S6B). The data thus far suggest that presentation of viral antigens on the thymic DCs and TECs seems to play a key role in inducing clonal deletion of viral antigen-reactive thymocytes.

### FV-specific CD8^+^ T cells, if recruited during the chronic phase of infection, can differentiate into functional memory CD8^+^ T cells

In the case of other models of chronic infection in which the pathogens do not disseminate to, or disappear from, the thymus [e.g. polyoma virus infection or more than 30 days after lymphocytic choriomeningitis virus (LCMV) clone 13 infection], newly recruited virus-specific CD8^+^ T cells can be primed and differentiate into functional memory CD8^+^ T cells [5], [6]. Therefore, we hypothesized that if FV-specific CD8^+^ T cells were generated in animals chronically infected with FV, such recent thymic emigrants (RTEs) may be able to undergo a process of post-thymic maturation, and then be primed and differentiate into antiviral CD8^+^ T cells with a superior functional capacity as compared to the severely exhausted memory CD8^+^ T cells in the periphery. To test this possibility, we investigated if the immune environments in chronically infected animals influence the process of post-thymic maturation in the periphery. To this end, we used *Rag1*-GFP knock-in mice as a tool to distinguish RTEs based on the expression of GFP within peripheral T cells, and first analyzed total, mostly FV-nonreactive, CD8^+^ T cells since FV-infected mice lack the generation of FV-specific RTEs as shown above. Total numbers of GFP^+^CD8^+^ T cells in the spleens of FV-infected and age-matched uninfected mice were comparable at 6 weeks post infection (Figure S7A, B), confirming the unaffected T-cell generating function of the FV-infected thymus (Figure 4). Importantly, in the FV-infected mice, while a large proportion of GFP^−^CD8^+^ T cells showed the highly activated phenotype (CD44^hi^CD69^+^PD-1^hi^), GFP^+^CD8^+^ RTEs at 6 weeks post infection showed minimal signs of abnormal activation as they mostly remained CD44^lo^CD69^−^PD-1^lo^ (Figure S7C). Since GFP expression is reduced concurrently with Rag expression ceasing 2–3 weeks after TCR rearrangements (1–2 weeks after exiting from the thymus) [23], GFP^+^CD8^+^ T cells detected at 6 weeks post infection should have been generated after FV infection, and thus the above results on unactivated phenotypes indicate that the RTEs did not receive bystander inflammatory signals even in the chronically infected environment. A transient increase in the proportion of activated (CD44^hi^CD69^+^PD-1^hi^) CD8^+^ RTEs at the peak of infection (2 weeks post FV infection) also supports this idea (Figure S7C). At 6 weeks post infection, gradual loss (CD24) and gain (CD127 and Qa2) of surface antigen expression by CD8^+^ RTEs revealed a prototypic status of post-thymic maturation in FV-infected mice (Figure S6D) [23]. Furthermore, although CD8^+^ RTEs typically have only a weak capacity to produce cytokines [24], GFP^+^CD8^+^ T cells in FV-infected mice were capable of producing IFN-γ and IL-2 upon stimulation with anti-CD3 antibody at levels comparable with those in the uninfected mice (Figure S7E, F). These results indicate that chronic infection with FV has little, if any, impact on the post-thymic maturation of RTEs in general.

**Figure 7:**
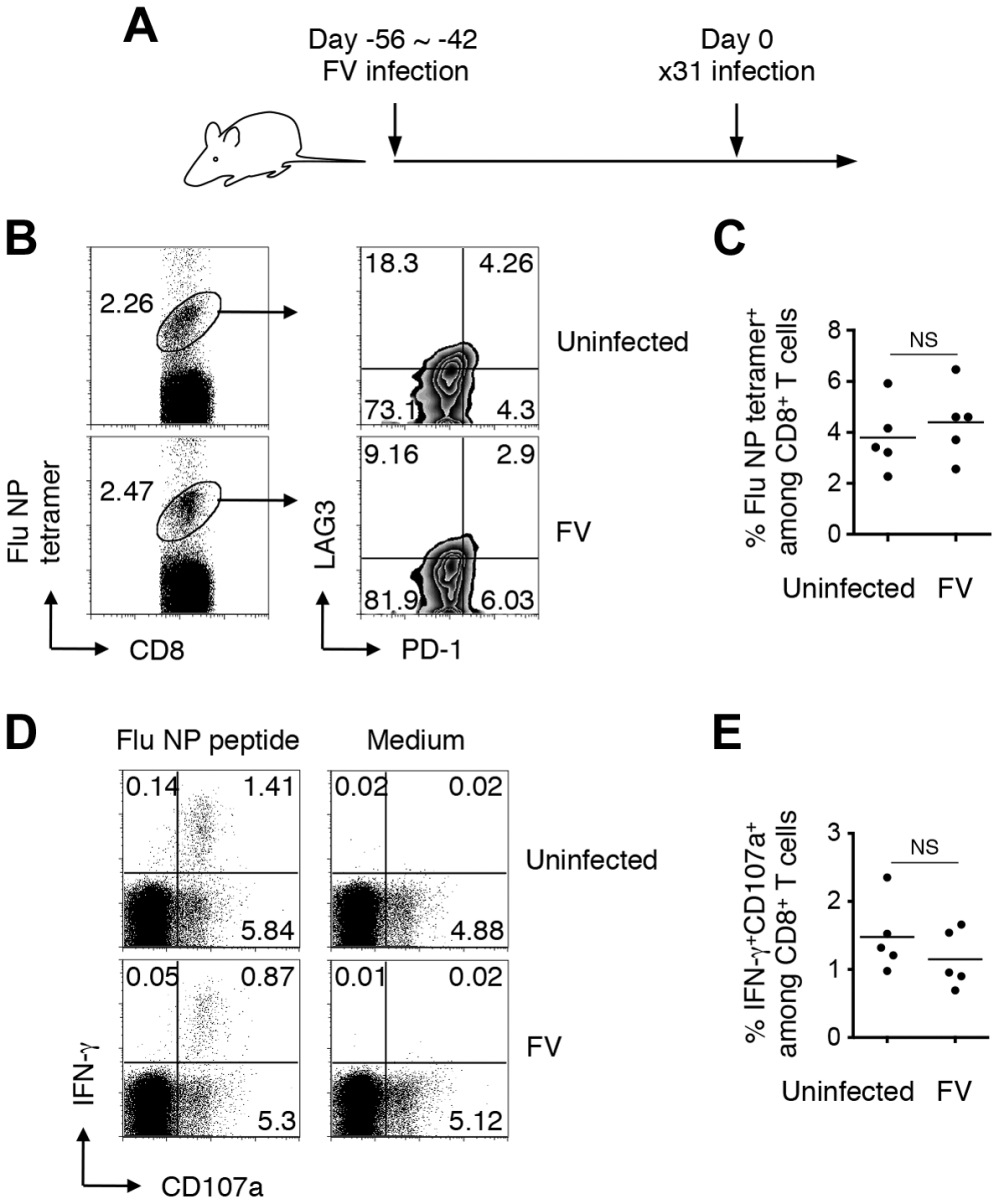
Generation of CD8^+^ T cell responses in mice chronically infected with FV. (A) FV-infected (6–8 weeks post infection) and age-matched uninfected mice were challenged i.n. with influenza virus ×31. (B, C) At day 11 after ×31 infection, splenocytes were isolated and stained with the indicated Abs and Flu NP_366–374_/D^b^ tetramer. (B) Shown are representative staining patterns for CD8 and the tetramer of CD8^+^ T cells, and LAG3 and PD-1 expression on tetramer^+^ cells. (C) Frequencies of NP_366–374_/D^b^ tetramer^+^ cells among CD8^+^ T cells in the spleen. (D, E) Fractions of cells were stimulated with either NP_366–374_ peptide or anti-CD3 Ab. The intracellular expression of IFN-γ and the surface expression of CD107a were measured by flow cytometry. (D) Shown are representative staining patterns for IFN-γ and CD107a of CD8^+^ T cells. (E) Frequencies of IFN-γ^+^CD107a^+^ cells among CD8^+^ T cells. Each symbol represents an individual mouse. Functionally competent influenza virus-specific CD8^+^ T cells were detected even at day 30 post challenge (data not shown). Average percentages of tetramer^+^ cells are not significantly different between the groups.

Nevertheless, it is possible that chronic infection with FV may create an immune suppressive environment that impairs the peripheral priming of CD8^+^ T cells. To examine this possibility we next asked whether naïve (post-thymically matured) CD8^+^ T cells could be adequately primed in animals chronically infected with FV. To do this, mice were infected with FV, and 6–8 weeks later were further infected with an FV-unrelated pathogen, influenza ×31 (Figure 7A). Eleven days post ×31 infection, FV-infected mice nevertheless developed robust anti-influenza CD8^+^ T cell responses detectable with MHC tetramer staining and antigen-specific IFN-γ production in association with the surface expression of CD107a (Figure 7). Importantly, levels of the influenza NP-specific CD8^+^ T cell responses in the FV-infected mice were as high as those in the uninfected mice, indicating that chronic FV infection had minimal impact on the induction of influenza-specific CD8^+^ T cell responses (Figure 7). Differentiation of functionally competent memory CD8^+^ T cells was also observed in FV-infected mice at a later stage of influenza virus infection (data not shown). Similar to the model shown in Figure S1, however, FV-specific CD8^+^ T cells generated in the same FV-infected animals were severely exhausted at any time-point examined in this experimental setting (data not shown). These results indicate that even in the situation where the functions of FV-specific CD8^+^ T cells were severely compromised (Figure S1), animals chronically infected with FV had no, if any, defects in mounting FV-unrelated CD8^+^ T cell responses.

**Figure 8:**
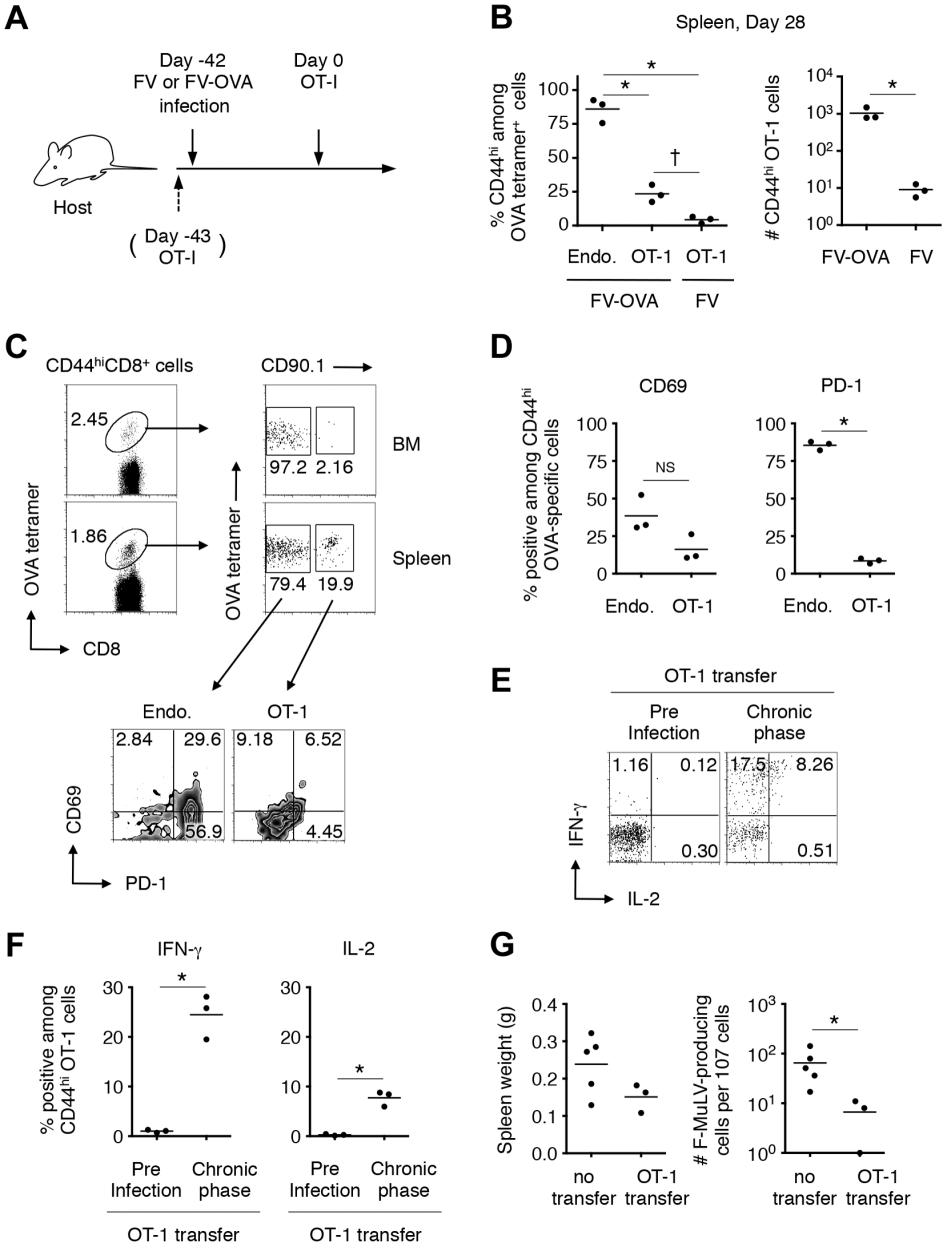
FV-specific CD8^+^ T cells can differentiate into functional memory CD8^+^ T cells if recruited during the chronic phase of infection. (A–D) B6AF_1_ mice were infected with either FV or FV-OVA. Six weeks later, FACS-sorted naïve (CD44^lo^) CD8^+^ T cells (1–2×10^7^) from (OT-1-Thy1.1× A/WySnJ)F_1_ mice were transferred i.v. Splenocytes and BM cells were isolated at 28 days post transfer and stained with the indicated Abs and OVA_257–264_/K^b^ tetramer. (B) Percentages of CD44^hi^ cells among OT-1 cells or endogenous OVA-specific CD8^+^ T cells, and actual numbers of CD44^hi^ OT-1 cells recovered from FV-OVA- or FV-infected mice. Averages between groups in the left panel were compared by one-way ANOVA with Tukey's multiple comparison test: *, *p*<0.001; †, *p*<0.05. Averages between FV-OVA and FV groups were compared by Welch's *t*-test: *, *p* = 0.047. (C) Representative staining patterns for PD-1 and CD69 among OT-1 cells or endogenous OVA-specific CD8^+^ T cells in the BM and spleen. (D) Expression of CD69 and PD-1 on CD44^hi^ OVA-specific CD8^+^ T cells in the spleen. Averages were compared for the two parameters between the endogenous and OT-1 cells: *, *p*<0.00001<α_2_ (0.05) = 0.0253 by student's *t* test with Bonferroni's correction for multiple comparisons. (E–F) A group of mice received 5×10^3^ OT-1 cells 1 day prior to FV-OVA infection as a control for CD8^+^ T cell responses that were primed by initial infection. Splenocytes were stimulated in vitro with OVA_257–264_/K^b^ peptide. Shown are intracellular expression levels of IFN-γ and IL-2 of CD44^hi^ OT-1 cells primed at the initial infection or during the chronic phase of infection. Averages were compared for the two parameters between the preinfection transfer and chronic phase groups: *, *p* = 0.012<α_2_ (0.05) = 0.0253 for IFN-γ and *p* = 0.013<α_2_ (0.05) = 0.0253 by Welch's *t*-test with Bonferroni's correction. (G) B6AF_1_ mice were infected with 5,000 focus-forming units of F-MuLV-OVA plus 2,000 SFFU of FV and naïve OT-1 cells (1×10^7^) were transferred 28 days after infection. Four weeks after transfer of naïve OT-1 cells, spleen weights were measured, and splenocytes were cocultured with *M. dunni* cells to enumerate F-MuLV infectious centers. Each symbol represents an individual mouse. *, significantly smaller in the numbers of infectious centers in comparison with those in non-transferred mice; *p* = 0.0357 by Mann-Whitney test for non-Gaussian distributions.

Based on the above observations that FV-unrelated CD8^+^ T cells can be activated in a chronically FV-infected environment, we next investigated whether FV-specific naïve CD8^+^ T cells recruited from FV-uninfected exogenous sources can be primed and differentiate into functional memory CD8^+^ T cells in the recipients during the chronic phase of infection. To do this, naïve (CD44^lo^) OT-1 cells were transferred into mice that had been infected chronically with either FV or FV-OVA (Figure 8A). As shown in Figure 8B, a small but significant proportion of transferred OT-1 cells were found to be primed in the presence of persistent OVA antigen expressed from FV-OVA at 4 weeks post transfer. As expected, the expression of PD-1 on the newly primed OT-1 cells was significantly lower than that on highly exhausted endogenous OVA-specific CD8^+^ T cells (Figure 8C, D), indicating that newly primed virus-specific CD8^+^ T cells were not exhausted. Interestingly, transferred OT-1 cells were rarely recruited to the BM (Figure 8C), a hot spot of persistent antigen presentation (Figure S1B). This might explain why newly primed OT-1 cells were not instantly exhausted. Slightly lower levels in the expression of the recent activation marker CD69 on OT-1 cells as compared to that on endogenous OVA-specific CD8^+^ T cells is probably due to the priming of naïve OT-1 cells needing professional APCs while endogenous, antigen-experienced OVA-specific CD8^+^ T cells can be reactivated by most virus-infected cells (Figure 8C, D). Intracellular cytokine staining of antigen-experienced (CD44^hi^) OT-1 cells revealed a high-level production of IFN-γ in response to OVA_257–264_ peptide stimulation, which was observed exclusively in newly primed OT-1 cells transferred in the chronic phase of infection, but not in OT-1 cells transferred prior to FV-OVA infection (thus, those primed in the early phase of infection) (Figure 8A, E, F). Moreover, newly primed OT-1 cells still retained the ability to produce IL-2, a function that disappeared primarily in the exhausted CD8^+^ T cells (Figure 8E, F) [25]. OT-1 cell transfer into FV-OVA-infected mice resulted in a slight reduction in spleen weights and a significant decrease in the numbers of infectious centers (Figure 8G), indicating that late-primed OT-1 cells can control chronic infection at least to some extent. Although we cannot predict from the above observation that newly recruited FV-specific CD8^+^ RTEs would control chronic infection, as the number of transferred OT-1 cells (1×10^7^) might be non-physiological, the above results clearly demonstrate that, despite the replication-competent component of the FV-OVA in the present experiment being a mixture of F-MuLV-OVA and wild-type F-MuLV, a significant proportion of infected erythroid cells expressed the OVA epitope. These results thus provide conclusive evidence that if FV-specific naïve CD8^+^ T cells are continuously recruited from the thymus even during the chronic phase of FV infection, cells newly primed with the persistent antigen can at least differentiate into the functional memory CD8^+^ T cells and may contribute to FV control. Overall, the above data indicate that FV-induced central tolerance inhibits the generation of virus-specific naïve CD8^+^ T cells that can otherwise be primed with FV antigens even in the chronic phase of infection, and the resultant lack of FV-reactive RTEs contributes to the observed lack of functional memory CD8^+^ T cells along with the exhaustion of antigen-experienced CD8^+^ T cells.

## Discussion

Recognition of self-antigens in the thymus is essential for the deletion of self-reactive thymocytes. In addition, silencing selection-escaped CD8^+^ T cells via abundant antigens and PD-1-mediated costimulation in the periphery is one of the mechanisms of peripheral tolerance, in addition to the suppression of self-reactive effector T cells by Tregs [26]. The data presented in our current and previous studies have demonstrated cunning strategies of murine retrovirus to evade anti-viral CD8^+^ T cell immunity by modulating both central and peripheral tolerance. During the acute phase of infection, massively expanded virus-infected PD-L1^hi^ erythroblasts disrupt the function of virus-specific effector CD8^+^ T cells in the periphery [21]. Then, the virus disseminates to the thymus and induces viral antigen presentation by DCs and TECs just as if self-antigens are present. In fact, endogenous retroviral antigens potentially cross-reactive to the exogenous retroviruses are known to be present in the thymus and shape T cell responses to the exogenous retroviruses by inducing the negative selection of low-avidity virus-reactive T cells [27]. On the other hand, the presentation of exogenously infecting viral antigens in the thymus inhibits subsequent production of virus-specific naïve CD8^+^ T cells as a source of functional memory CD8^+^ T cells in chronically infected animals as we have shown here for FV infection. Consequently, almost all virus-specific CD8^+^ T cells in FV-infected animals lack their effector functions. Although viral replication is controlled at low levels by the production of virus-neutralizing antibodies [8], mice chronically infected with FV are highly susceptible to challenge with FV-induced tumor cells against which CD8^+^ T cell response is required for effective elimination.

Induction of pathogen-specific central tolerance through thymic dissemination is not a unique feature of FV infection. It has been reported that neonatal infection with Gross murine leukemia virus (G-MuLV) or Moloney murine leukemia virus (Mo-MuLV) results in intrathymic viral replication that induces life-long immune nonresponsiveness to viral antigens [28], [29]. It should be noted, however, that when inoculated into adult mice, Mo-MuLV is rapidly eliminated and does not cause leukemia [30]. Interestingly, experiments on intrathymic (i.t.) inoculation revealed that G-MuLV infects predominantly thymic epithelial cells, while Mo-MuLV, which does not induce tolerance following i.t. inoculation in adult mice, favors mature T cells as targets of infection in the thymus [28], [31], [32]. In the case of adult infection, as mentioned above, LCMV clone 13 is known to disseminate to the thymus and induce virus-specific central tolerance [33]–[35]. Recently, some species of *Mycobacteria* have also been reported to induce bacteria-specific T cell tolerance via thymic dissemination [36], [37]. Since some extent of functional pathogen-specific CD8^+^ T cells remain in these chronically infected animals, loss of pathogen-specific RTEs does not make a significant impact on ongoing infections [38]. Contrarily, FV infection leads to almost complete loss of functional virus-specific CD8^+^ T cells in the periphery [21]. Furthermore, because functional antigen-specific CD8^+^ T cells in the periphery re-enter the thymus and can delete infected APCs to abrogate central tolerance [33], [34], [39], early and overall exhaustion of functional FV-specific CD8^+^ T cells in the periphery may extend viral persistency in the thymus due to ineffective viral clearance in this organ.

A question that remains is how FV initially disseminates to the thymus. The finding that CD44^hi^ DN thymocytes lack the expression of viral proteins indicates that BM-originated common lymphoid progenitor cells are unlikely to be an active transporter of the virus to the thymus. In the steady state, it has been demonstrated that conventional DCs, as well as plasmacytoid DCs in the blood, transport peripheral self-antigens to the thymus and initiate central tolerance [40], [41]. In pathological conditions, however, the activation/maturation process strongly inhibits the migration of peripheral DC populations to the thymus by modulating their chemokine receptor expression [40], [41], thus preventing the unfavorable induction of acquired tolerance to the invading pathogens. However, it has become evident that in some cases, such as infections with *Mycobacteria* and highly pathogenic influenza virus, pathogen-infected DCs of an extrathymic origin can transport the infectious microorganisms to the thymus, resulting in thymic dissemination and subsequent thymic dysfunctions [36], [42]. Although we do not exclude the possibility that this could be the case in FV infection, as gp70 expression was observed on extrathymic DCs in the thymus, we could not detect the production of infectious virions from any thymic DC population even at the peak of infection. Alternatively, because prior exposure to antigen enhances the migration of mature T cells to the thymus, activated T cells that are infected with the virus in the periphery, if there are any, might be another potential source in transporting the virus into the thymus. However, faint expression of gp70 on CD44^hi^, CD4 SP and CD8 SP populations in FV-infected thymus makes this unlikely. Since F-MuLV can infect and replicate in endothelial cells at high levels, and the virus particles can be found in a subendothelial location of blood vessels [43], [44], it is reasonable to postulate that FV disseminates to the thymus through the replication in the endothelial cells and subsequent production of viral particles to the parenchymal side, rather than via the migration of virus-infected hematopoietic cells into the organ.

Another issue to be considered is whether thymic infection with FV results in the generation of Tregs. It has become clear that thymocytes that display TCRs with higher affinity (but much lower than that induces negative selection) for thymic MHC/self-peptide ligands can develop into Tregs [45], [46]. Thus, not only the induction of negative selection, but the generation of naturally occurring Tregs (nTregs) harboring TCRs reactive with non-self antigen could also be induced by thymic infection with pathogens [47]. As an increase in the number of Tregs in lymphoid organs has long been recognized in FV-infected animals [16]–[18], [48], dissemination of the virus into the thymus might be one of the factors that cause the increase of Tregs. In fact, the observed expansion of Tregs peaked around 2 weeks post FV infection [16], approximately the same time-point that viral antigen expression in the thymus was peaking. In the recent study, moreover, adoptive transfer of FoxP3^−^ CD4^+^ T cells and tracking neuropilin-1 expression as a marker of nTregs revealed that Tregs that expanded after FV infection originated from thymus-derived nTregs but not from FoxP3^−^ conventional CD4^+^ T cells in the periphery [48]. Importantly, however, it was suggested that Tregs that expanded after FV infection lack the expression of TCRs specific for FV antigen [48], [49]. Therefore, viral antigen expression in the thymus may preferentially contribute to the deletion of FV-reactive conventional T cells rather than the induction of nTregs carrying FV-reactive TCRs.

In summary, the loss of antigen-specific RTE production induced by thymic infection is a unique and powerful evasion strategy from antiviral CD8^+^ T cell responses, especially when the functions of virus-specific CD8^+^ T cells in the periphery are concomitantly severely compromised during the chronic phase of infection. Similar synergistic negative consequences could be induced in chronic infection with other thymotropic viruses such as HIV, although mechanisms of thymic failure may differ. In fact, HIV is known to cause thymotoxic infection that prevents adequate T cell generation [50], while FV infection does not affect thymocyte differentiation in general. There is no doubt that reinvigoration of exhausted virus-specific CD8^+^ T cells in the periphery is a primary therapeutic target as we have shown in the case of acute FV infection [21]. However, as successful recovery from AIDS under anti-retroviral therapy is largely owing to the thymus-driven immune reconstitution, careful monitoring of the thymic function would be required in cases of thymotropic virus infection.

## Materials and Methods

### Ethics statements

The studies utilizing laboratory animals were carried out in strict accordance with the Act on Welfare and Management of Animals of the Government of Japan and the Regulations for the Care and Use of Laboratory Animals of Kinki University. The protocol for the present study was approved by the Institutional Animal Experimentation Committee of Kinki University Faculty of Medicine (Permit Number: KAME-20-066). All surgery was performed under sodium pentobarbital anesthesia, and all efforts were made to minimize suffering.

### Viruses, mice, cells and infection/injection

An original stock of B-tropic FV complex without contamination of lactate dehydrogenase-elevating virus was kindly provided by Kim Hasenkrug (NIH, NIAID, Rocky Mountain Laboratories, Hamilton, MT). FV was expanded, stored, and titered as previously described [51]. An infectious molecular clone of F-MuLV, FB29, was prepared from culture supernatant of chronically infected *Mus dunni* cells as previously described [51]. A plasmid harboring the permutated FB29 cDNA was kindly provided by Marc Sitbon (Institut de Génétique Moléculaire de Montpellier, Montpellier, France). To insert the OVA epitope (SIINFEKL) into the C-terminus of the F-MuLV envelope protein in-frame, a pair of PCR primers that hybridize with the 3′ region of the F-MuLV genome and harbor the OVA sequence were used along with the above plasmid as the template (Figure S4B). Infectious F-MuLV-OVA was produced by transfecting *Mus dunni* cells with the mutant plasmid. Influenza virus A/HK-×31 was provided by David L. Woodland (Keystone Symposia, Silverthorne, CO). FBL3 is an F-MuLV-induced leukemia of B6 origin that expresses FV-related antigens [21]. EL-4 (a T cell lymphoma line derived from B6 mice) and E.G7 (EL-4 cells expressing OVA protein) were purchased from ATCC (Manassas, VA). C57BL/6NCrSlc (B6) mice were purchased from Japan SLC, Inc. (Shizuoka, Japan). A/WySnJ, B6.PL-*Thy1* ^a^/CyJ (Thy1.1; CD90.1^+^) and B6.SJL-*Ptprc* ^a^ *Pepc^b^*/BoyJ (Pep/Boy; CD45.1^+^) mice were purchased from The Jackson Laboratory (Bar Harbor, ME). OT-1 TCR transgenic mice with the B6 background (OT-1 mice) were kindly provided by Miyuki Azuma (Tokyo Medical and Dental University, Tokyo, Japan) with the permission of William R. Heath (University of Melbourne, Victoria Australia) [52]. OT-1 and Thy1.1 mice were crossed and F_2_ progeny mice were selected for OT-1 TCR and Thy1.1 homozygosity by using monoclonal antibody (mAb) specific for mouse TCR Vα2, CD90.1 and CD90.2. OT-1-Thy1.1 mice thus obtained were >98% Vα2^+^ among CD8^+^ T cells, CD90.1^+^ and CD90.2^−^. (OT-1-Thy1.1× A/WySnJ)F_1_ mice were confirmed to be OT-1 TCR^+^ and Thy1.1^+^ and used in this study, and T cells separated from the above F_1_ mice are termed OT-1 T cells in the text. *Rag1*-GFP knock-in mice with the B6 background have been described [53]. Animals were housed and bred in the Experimental Animal Facilities at Kinki University Faculty of Medicine under specific pathogen-free conditions. Due to a mutation in the intron of the *Stk* gene, B6 mice lack the expression of sf-Stk, and are resistant to SFFV-induced expansion of virus-infected erythroblasts and resultant splenomegaly [11], [12]. As this erythroid cell expansion induced during the early phase of infection has been shown to be the major cause of severe exhaustion of virus-specific CD8^+^ T cells [21], we used B6AF_1_ mice that express sf-Stk in this study. Both male and female B6AF_1_ mice, 6 to 10 weeks old, were infected intravenously (i.v.) either with 1,000 spleen focus-forming units (SFFU) of FV or with 2,000 focus-forming units of F-MuLV-OVA plus 1,000 SFFU of FV (termed FV-OVA). For tumor rejection experiments, mice were injected subcutaneously (s.c.) with 5×10^6^ of FBL3 or EL-4 cells. Some experimental mice were challenged intranasally (i.n.) with 300 egg infective dose 50 (EID_50_) of ×31.

### Tissue harvest and flow cytometry

Mice were sacrificed at the indicated time points and single-cell suspensions from each tissue were obtained by straining through nylon mesh, depleted of erythrocytes in buffered ammonium chloride, and panned on goat anti-mouse IgG (H+L) (KPL, Gaithersburg, MD) for tetramer staining [21]. Live-cell counts were determined by trypan blue exclusion. Isolated cells were incubated with anti-CD16/32 (BD Biosciences, San Diego, CA) for 15 min on ice to prevent test antibodies (Abs) from binding to Fc receptors, and then stained either with APC-conjugated F-MuLV gag_75–83_/D^b^ tetramer, influenza virus nucleoprotein (NP)_366–374_/D^b^ tetramer, or OVA_257–264_/K^b^ tetramer for 1 h at room temperature. All tetramers were generated at the Trudeau Institute Molecular Biology Core (Saranac Lake, NY). Tetramer-labeled cells were then washed and stained with FITC-, PE-, PerCP/Cy5.5-, or APC-conjugated antibodies for 30 min on ice. Antibodies reactive to the following molecules were purchased from BioLegend (San Diego, CA): CD3, CD4, CD8, CD11b, CD11c, CD19, CD44, CD69, CD80, CD90.1, CD90.2, PD-1, LAG-3, B220, Ly51, TCR Vα2, and EpCAM. Purified anti-F-MuLV envelope gp70 mAb, clone 720 [43], anti-F-MuLV gag p15 mAb, clone 34 [54], or mouse IgG_1_ isotype control, clone 1B7, were labeled with biotin by using NHS-PEG_4_-Biotin (Thermo Scientific, Waltham, MA) or with FITC by using Fluorescein Labeling Kit-NH_2_ (Dojindo Molecular Technologies, Inc., Rockville, MD) as described [55]. Labeled streptavidin was purchased from BioLegend. Samples were run on a FACSCalibur or a FACSAria (BD Biosciences). All data were analyzed with FlowJo software (Tree Star, Ashland, OR).

### *In vitro* restimulation and intracellular cytokine staining

Splenocytes isolated from infected mice as described above were seeded in 96-well plates at a concentration of 1×10^6^ cells per well. Cells were incubated in the presence of Alexa488-conjugated anti-CD107a (BioLegend), monensin A (BioLegend) and either F-MuLV gag_75–83_ peptide (5 µM), influenza NP_366–374_ peptide (5 µM), OVA_257–264_ peptide (5 µM), or anti-CD3 (4 µg/ml) (eBiosciences, San Diego, CA) for 2 h at 37°C; brefeldin A (50 µg/ml) was then added, and the incubation was continued for an additional 4 h. Surface staining for CD8 was performed as described above, and the cells were fixed and permeabilized with the Cytofix Cytoperm kit (BD Bioscience). For the detection of intracellular IFN-γ and IL-2, the cells were incubated for 15 min in the Perm/Wash buffer followed by incubation in the same buffer with anti-IFN-γ (BioLegend) and anti-IL-2 (BD Bioscience) for 1 h; the cells were then washed and analyzed as described above. To correct for background variations between experiments, we subtracted the percentage of IFN-γ^+^, IL-2^+^ or CD107a^+^ cells among CD8^+^ T cells without stimulation from the percentage of IFN-γ^+^, IL-2^+^ or CD107a^+^ cells following peptide stimulation, for each individual mouse.

### Infectious center assays

Cells prepared from each tissue were serially diluted and plated in duplicate at concentrations between 1×10^2^ and 1×10^6^ cells onto monolayers of *Mus dunni* cells. After being washed and fixed with methanol on the second day of coculturing, cells were stained with biotinylated mAb 720, and F-MuLV-infected foci were visualized by using Elite ABC Kit (Vector Laboratories, Burlingame, CA) as described [21].

### Immunohistochemistry

The thymuses from naïve or FV-infected animals were harvested, embedded in OCT compound (Sakura Finetek, Tokyo Japan), and frozen in liquid nitrogen. Frozen sections (6 µm) were fixed with 4% paraformaldehyde for 10 min. After quenching endogenous biotin activity, sections were stained with combinations of PE-conjugated anti-CD11c, anti-ER-TR4 (specific for cortical thymic epithelial cells; cTECs), anti-ER-TR5 (specific for medullary thymic epithelial cells; mTECs) [56], biotin-conjugated anti-F-MuLV gag p15, clone 690 [57], and biotin-conjugated anti-F-MuLV gag p30, clone R18-7 [54]. Secondary antibodies, PE-conjugated anti-rat IgM, PE-conjugated anti-rat IgG, and AF488-conjugated streptavidin were used to visualize cTEC, mTEC and FV antigens, respectively. All frozen sections were observed and images recorded with a fluorescence microscope (BioZero; Keyence Japan).

### Thymectomy and thymus transplantation

Day 5 neonatal pups were thymectomized. Five weeks later, mice were injected with purified anti-CD8 (2.43) and anti-CD4 (GK1.5) antibodies to deplete T cells in the periphery. A thymic lobe from FV-infected (2–3 weeks post infection) or age-matched uninfected donors was grafted under the kidney capsule. Six weeks later, mice were challenged with either FBL3 or infected with ×31.

### Fetal thymus organ culture (FTOC)

Thymic stromal cells of B6AF_1_ mice were prepared from E15.5 fetal thymus lobes that were cultured for 5 days in the presence of 2-deoxyguanosine [22]. OT-1 DP thymocytes were sorted from adult (OT-1-Thy1.1× A/WySnJ)F_1_ mice. Thymocytes, thymic DCs or TECs from B6AF_1_ mice infected with FV-OVA 21 days prior to the preparation were sorted as third populations. CD45-negative cells were enriched by depleting CD90^+^ cells with a magnetic cell sorter (BD Bioscience) prior to sorting of thymic DC and TEC populations. Thymic stromal cells (2–3×10^5^) and OT-1 DP thymocytes (3–5×10^5^) were reaggregated and organ-cultured for 4 days in the presence of each third population (0.5–2×10^5^) as described [22].

### OT-1 cell transfer

Splenocytes from (OT-1-Thy1.1× A/WySnJ)F_1_ mice were enriched for CD8^+^ cells by negative selection using Ab-conjugated micromagnetic beads as described [58]. Cells were then stained with anti-CD44-FITC, and CD44^lo^ cells were sorted using FACSVantage cell sorter with DIVA enhancement software (BD Bioscience). A total of 1–2×10^7^ cells were transferred into recipient B6AF_1_ mice infected with either FV or FV-OVA 42 days prior to the cell transfer.

### Real-Time PCR

Total RNA was purified from the spleen, BM, and thymus of FV-infected mice using RNeasy Blood & Tissue kit (Qiagen, Hilden, Germany) and cDNA synthesis was performed by using PrimeScript RT reagent Kit (Takara Bio Inc., Shiga, Japan). The viral DNA fragments were amplified from 0.5 µg or 1 µg of total cDNA and were quantified using Platinum Quantitative PCR SuperMix-UDG with ROX (Life Technologies, Carlsbad, CA) and a Prism 7900HT Real-Time PCR system (Life Technologies). PCR primers and TaqMan probes for the differential detection of F-MuLV and SFFV cDNAs were designed on the *env* portion of each provirus: primers 5′-AAGTCTCCCCCCGCCTCTA-3′ and 5′-AGTGCCTGGTAAGCTCCCTGT-3′, and a FAM-labeled probe 5′-ACTCCCACATTGATTTCCCCGTCC-3′ for the detection of F-MuLV, and primers 5′-TCTAACCTCACCAACCCTGAT-3′ and 5′-TTTTAGGGCAATGGTATGATTAAAATAA-3′, and a FAM-labeled probe 5′-CCTAGTGTCTGGACCCCCCTATTACGAGG-3′ for the detection of SFFV [59]. After initial incubations at 50°C for 2 min and 95°C for 10 min, 40 cycles of amplification were carried out at 95°C for 30 sec and at 58°C for 1 min. A TaqMan rodent GAPDH control reagent (Life Technologies) was used as an internal control. Standard curves obtained by using plasmids containing the *env* gene of each virus as templates were linear over a range of 10–10^6^ copies in the above reaction.

### Statistical analysis

Statistical analyses were performed using Prism software (GraphPad Software, Inc., San Diego, CA). Methods of comparison and corrections for multiple comparisons are indicated in each relevant figure legend.

### Accession numbers

Friend murine leukemia virus FB29 complete genome (accession number: Z11128).

## Supporting Information

Figure S1 **Virus-specific CD8^+^ T cells in mice chronically infected with FV are non-responsive to the viral antigen.** (A) FV-infected (6–8 weeks post infection) and age-matched uninfected mice were injected s.c. with FBL3 tumor cells (5×10^6^). (B) At day 42–56 after infection (Before tumor injection) cells purified from the BM (upper panels) and spleen (lower panels) were stained with the indicated Abs and F-MuLV gag_75–83_/D^b^ tetramer. Shown are representative staining patterns for CD8 and the tetramer of CD8^+^ T cells, and PD-1, CD69, LAG-3 and Tim-3 on tetramer^+^ cells. (C) At day14 after FBL3 injection, splenocytes were isolated and stained with the indicated Abs and F-MuLV gag_75–83_/D^b^ tetramer. Shown are representative staining patterns for CD8 and the tetramer of CD8^+^ T cells. (D) Fractions of spleen cells were stimulated with the gag_75–83_ peptide or cultured without stimulation (Medium). The intracellular expression of IFN-γ and the surface expression of CD107a were measured by flow cytometry. Shown are representative staining patterns for intracellular IFN-γ and surface CD107a expression of stimulated and unstimulated CD8^+^ T cells. Tumor sizes (E) and host survival (F) are shown for uninfected (upper panels) and FV-infected (lower panels) animals (*n* = 6–11). Note that lines with tumor size zero in panel E include multiple individuals. Survival curves were compared between the uninfected and FV-infected groups by Mantel-Cox log-rank test: *, *p* = 0.0001.
(DOC)

Figure S2 **Viral antigen expression in each cell population in the thymus after FV or F-MuLV infection.** B6AF_1_ or B6 mice were infected with various doses of FV or F-MuLV. At day 14 after infection, cells in the thymus were isolated and stained with the indicated Abs. Shown are representative staining patterns and gating protocols of thymocytes. Data are representative of two independent experiments with essentially similar results.
(DOC)

Figure S3 **Immunohistochemistry of the thymuses from FV-infected mice.** Experiments were performed as described for Figure 3C. Arrowheads indicate cells doubly positive for the indicated cell surface marker and the viral antigen. Representative view-fields from those shown here are presented in Figure 3C.
(DOC)

Figure S4 **Spleen weights and gp70 expression on B cells and erythroblasts after thymic transplantation.** Experiments were performed as described for Figure 5. (A) Spleen weights were measured at day 14 after transplantation. (B) At day 14 after transplantation, splenocytes were isolated and stained with the indicated antibodies. Shown are representative staining patterns for gp70 on CD19^+^ and Ter119^+^ cells.
(DOC)

Figure S5 **Construction of F-MuLV-OVA.** (A) Schematic representation of the F-MuLV-OVA construct. A synthetic oligonucleotide encoding the SIINFEKL epitope was inserted in-frame at the 3′ end of the *env* gene (B) Detailed strategy for the generation of F-MuLV-OVA. Oligonucleotide primers harboring the OVA epitope sequence and hybridizing with the F-MuLV genome at the end of the *env* gene were used for PCR-based mutagenesis with the permutated molecular clone of F-MuLV as the template. F-MuLV genome sequence and base numbers shown are according to the database information (Z11128). The vertical arrow indicates the site of cleavage that generates fusogenic TM protein and R peptide [60]. (C) Splenocytes from naïve B6AF_1_ mice were infected in vitro with either F-MuLV or F-MuLV-OVA. Cells were then cocultured with CD8^+^ T cells purified from (OT-1-Thy1.1× A/WySnJ)F_1_ mice (OT-1 cell). Shown are representative histograms for CD69 expression on OT-1 cells.
(DOC)

Figure S6 **FACS profiles of cells from FTOC.** Experiments were performed as described for Figure 6. Either tumor cells (A) or thymic cell populations purified from FV-OVA-infected mice (B) were used as the third population. Shown are representative dot plots of positive control settings (A) and experimental settings (B).
(DOC)

Figure S7 **Post-thymic maturation of CD8^+^ RTEs in mice chronically infected with FV.** (*Rag1*-GFP×A.WySnJ)F_1_ mice were infected with 1,000 SFFU of FV. Splenocytes were isolated at the indicated time-points and stained with the indicated Abs. Shown are dot plots for GFP expression among CD4^+^ and CD8^+^ T cells at day 42 after infection (A), and actual numbers of GFP^+^CD8^+^ T cells in either FV-infected or age-matched naïve animals (B). No significant difference was observed between the groups. (C–D) Shown are representative staining patterns for PD-1, CD69 and CD44 of GFP^+^CD8^+^ T cells or GFP^−^CD8^+^ T cells (C), and for CD24, Qa2 and CD127 of GFP^−^, GFP^lo^ or GFP^hi^ cells (D). (E–F) Splenocytes were stimulated *in vitro* with anti-CD3 Ab. The intracellular expression of IFN-γ and IL-2 were then measured by flow cytometry. Shown are representative staining patterns for IFN-γ and CD107a of GFP^+^CD8^+^ T cells (E), and frequencies of IFN-γ^+^ cells and IL-2^+^ cells among GFP^+^CD8^+^ T cells (F). Each symbol represents an individual mouse. Average percentages were compared between uninfected and FV-infected groups by two-way ANOVA with Bonferroni's corrections for multiple comparisons, and no significant difference was detected. Data are representative of two independent experiments with essentially equivalent results.
(DOC)
