# Supplementary material for: Infection of Adult Thymus with Murine Retrovirus Induces Virus-Specific Central Tolerance That Prevents Functional Memory CD8+ T Cell Differentiation
Source: PLoS Pathog. 2014 Mar 20;10(3):e1003937. doi: 10.1371/journal.ppat.1003937 (PMC3961338; doi:10.1371/journal.ppat.1003937)
Supplement: Figure S1 — Virus-specific CD8+ T cells in mice chronically infected with FV are non-responsive to the viral antigen. (A) FV-infected (6–8 weeks post infection) and age-matched uninfected mice were injected s.c. with FBL3 tumor cells (5×10^6^). (B) At day 42–56 after infection (Before tumor injection) cells purified from the BM (upper panels) and spleen (lower panels) were stained with the indicated Abs and F-MuLV gag75–83/Db tetramer. Shown are representative staining patterns for CD8 and the tetramer of CD8+ T cells, and PD-1, CD69, LAG-3 and Tim-3 on tetramer+ cells. (C) At day14 after FBL3 injection, splenocytes were isolated and stained with the indicated Abs and F-MuLV gag75–83/Db tetramer. Shown are representative staining patterns for CD8 and the tetramer of CD8+ T cells. (D) Fractions of spleen cells were stimulated with the gag75–83 peptide or cultured without stimulation (Medium). The intracellular expression of IFN-γ and the surface expression of CD107a were measured by flow cytometry. Shown are representative staining patterns for intracellular IFN-γ and surface CD107a expression of stimulated and unstimulated CD8+ T cells. Tumor sizes (E) and host survival (F) are shown for uninfected (upper panels) and FV-infected (lower panels) animals (n = 6–11). Note that lines with tumor size zero in panel E include multiple individuals. Survival curves were compared between the uninfected and FV-infected groups by Mantel-Cox log-rank test: *, p = 0.0001. (DOC) [file ppat.1003937.s001.doc]

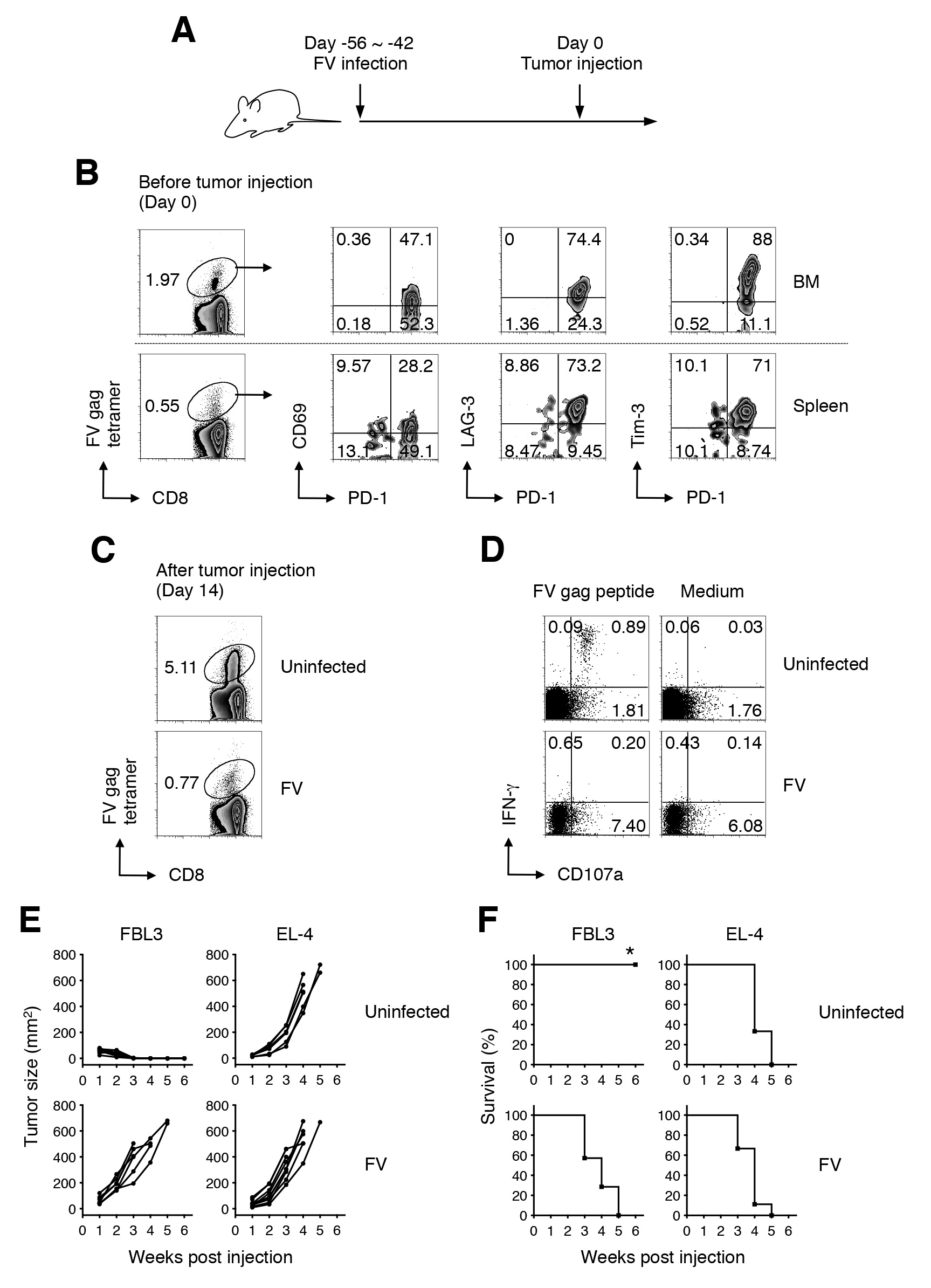
**Figure S1. Virus-specific CD8^+^ T cells in mice chronically infected with FV are non-responsive to the viral antigen.** (A) FV-infected (6-8 weeks post infection) and age-matched uninfected mice were injected s.c. with FBL3 tumor cells (5 × 10^6^). (B) At day 42-56 after infection (Before tumor injection) cells purified from the BM (upper panels) and spleen (lower panels) were stained with the indicated Abs and F-MuLV gag_75-83_/D^b^ tetramer. Shown are representative staining patterns for CD8 and the tetramer of CD8^+^ T cells, and PD-1, CD69, LAG-3 and Tim-3 on tetramer^+^ cells. (C) At day14 after FBL3 injection, splenocytes were isolated and stained with the indicated Abs and F-MuLV gag_75-83_/D^b^ tetramer. Shown are representative staining patterns for CD8 and the tetramer of CD8^+^ T cells. (D) Fractions of spleen cells were stimulated with the gag_75-83_ peptide or cultured without stimulation (Medium). The intracellular expression of IFN-γ and the surface expression of CD107a were measured by flow cytometry. Shown are representative staining patterns for intracellular IFN-γ and surface CD107a expression of stimulated and unstimulated CD8^+^ T cells. Tumor sizes (E) and host survival (F) are shown for uninfected (upper panels) and FV-infected (lower panels) animals (*n* = 6 - 11). Note that lines with tumor size zero in panel E include multiple individuals. Survival curves were compared between the uninfected and FV-infected groups by Mantel-Cox log-rank test: *, *p* = 0.0001.
