# Supplementary material for: Infection of Adult Thymus with Murine Retrovirus Induces Virus-Specific Central Tolerance That Prevents Functional Memory CD8+ T Cell Differentiation
Source: PLoS Pathog. 2014 Mar 20;10(3):e1003937. doi: 10.1371/journal.ppat.1003937 (PMC3961338; doi:10.1371/journal.ppat.1003937)
Supplement: Figure S2 — Viral antigen expression in each cell population in the thymus after FV or F-MuLV infection. B6AF1 or B6 mice were infected with various doses of FV or F-MuLV. At day 14 after infection, cells in the thymus were isolated and stained with the indicated Abs. Shown are representative staining patterns and gating protocols of thymocytes. Data are representative of two independent experiments with essentially similar results. (DOC) [file ppat.1003937.s002.doc]

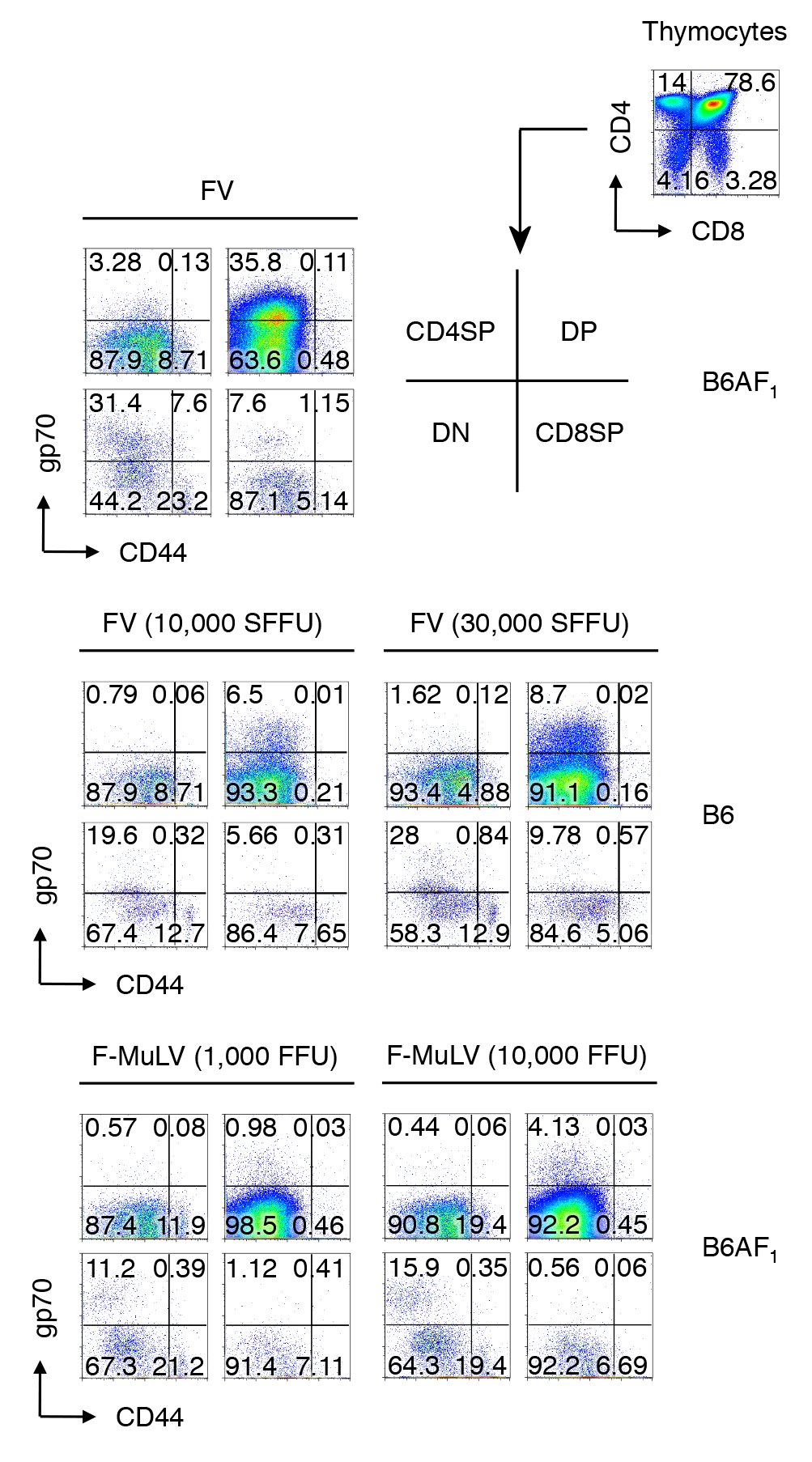


**Figure S2. Viral antigen expression in each cell population in the thymus after FV or F-MuLV infection.** B6AF_1_ or B6 mice were infected with various doses of FV or F-MuLV. At day 14 after infection, cells in the thymus were isolated and stained with the indicated Abs. Shown are representative staining patterns and gating protocols of thymocytes. Data are representative of two independent experiments with essentially similar results.
