# Supplementary material for: Infection of Adult Thymus with Murine Retrovirus Induces Virus-Specific Central Tolerance That Prevents Functional Memory CD8+ T Cell Differentiation
Source: PLoS Pathog. 2014 Mar 20;10(3):e1003937. doi: 10.1371/journal.ppat.1003937 (PMC3961338; doi:10.1371/journal.ppat.1003937)

**Figure S3. Immunohistochemistry of the thymuses from FV-infected mice.** Experiments were performed as described for Figure 3C. Arrowheads indicate cells doubly positive for the indicated cell surface marker and the viral antigen. Representative view-fields from those shown here are presented in Figure 3C.


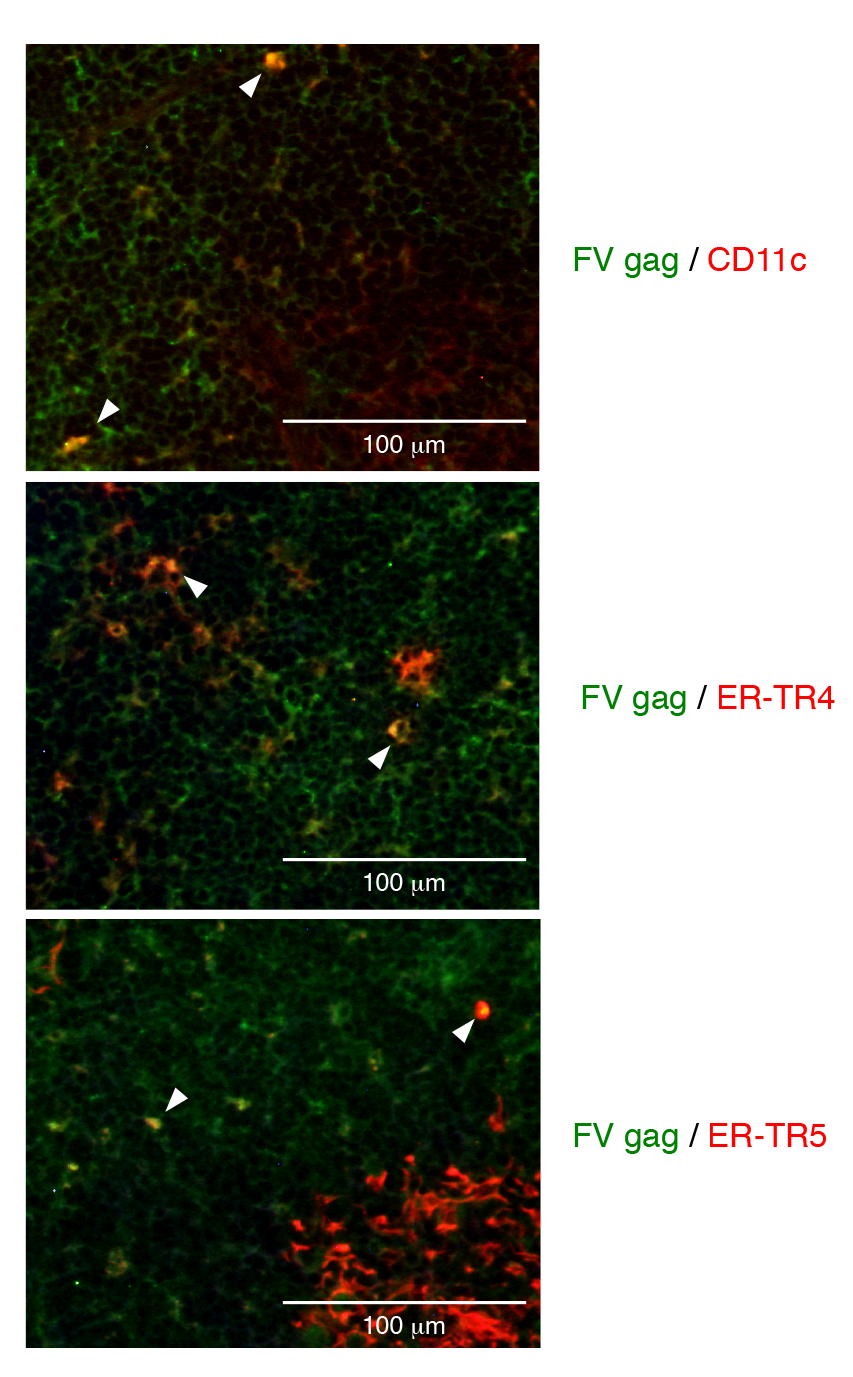

Supplement: Figure S3 — Immunohistochemistry of the thymuses from FV-infected mice. Experiments were performed as described for Figure 3C. Arrowheads indicate cells doubly positive for the indicated cell surface marker and the viral antigen. Representative view-fields from those shown here are presented in Figure 3C. (DOC) [file ppat.1003937.s003.doc]
