# Supplementary material for: Infection of Adult Thymus with Murine Retrovirus Induces Virus-Specific Central Tolerance That Prevents Functional Memory CD8+ T Cell Differentiation
Source: PLoS Pathog. 2014 Mar 20;10(3):e1003937. doi: 10.1371/journal.ppat.1003937 (PMC3961338; doi:10.1371/journal.ppat.1003937)
Supplement: Figure S4 — Spleen weights and gp70 expression on B cells and erythroblasts after thymic transplantation. Experiments were performed as described for Figure 5. (A) Spleen weights were measured at day 14 after transplantation. (B) At day 14 after transplantation, splenocytes were isolated and stained with the indicated antibodies. Shown are representative staining patterns for gp70 on CD19+ and Ter119+ cells. (DOC) [file ppat.1003937.s004.doc]

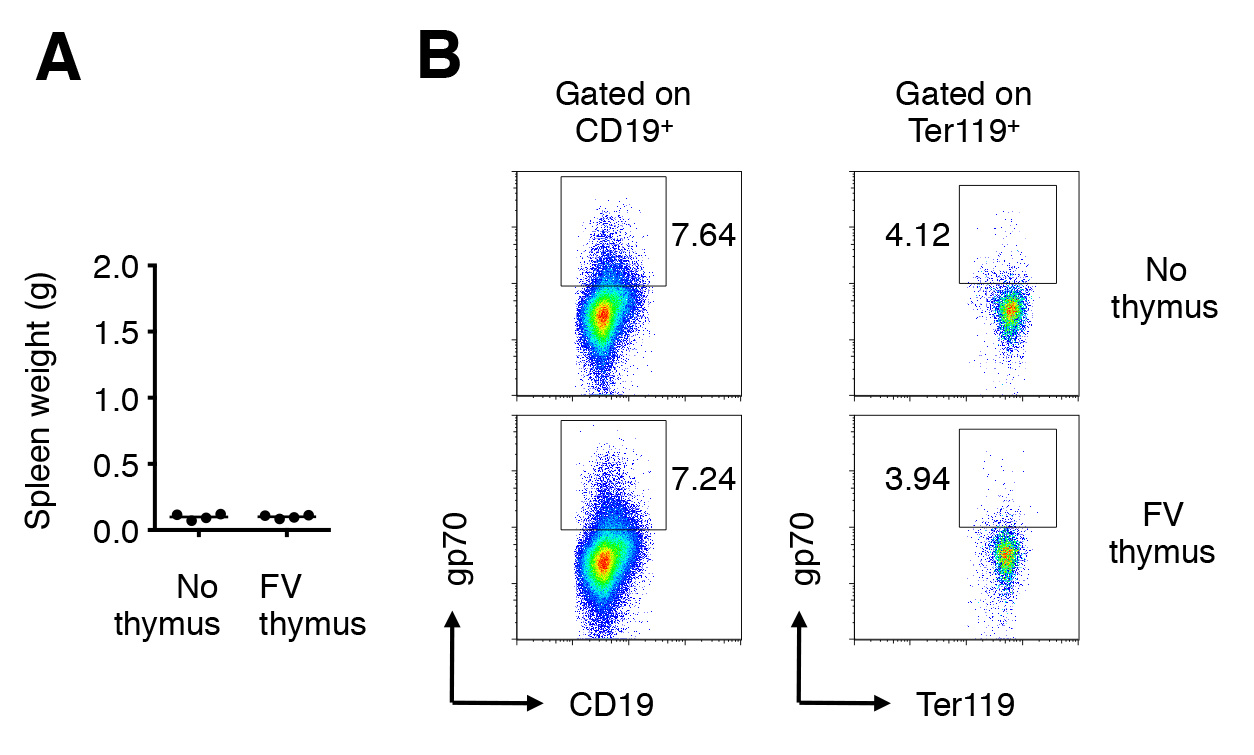


**Figure S4. Spleen weights and gp70 expression on B cells and erythroblasts after thymic transplantation.** Experiments were performed as described for Figure 5. (A) Spleen weights were measured at day 14 after transplantation. (B) At day 14 after transplantation, splenocytes were isolated and stained with the indicated antibodies. Shown are representative staining patterns for gp70 on CD19^+^ and Ter119^+^ cells.
