# Supplementary material for: Infection of Adult Thymus with Murine Retrovirus Induces Virus-Specific Central Tolerance That Prevents Functional Memory CD8+ T Cell Differentiation
Source: PLoS Pathog. 2014 Mar 20;10(3):e1003937. doi: 10.1371/journal.ppat.1003937 (PMC3961338; doi:10.1371/journal.ppat.1003937)
Supplement: Figure S5 — Construction of F-MuLV-OVA. (A) Schematic representation of the F-MuLV-OVA construct. A synthetic oligonucleotide encoding the SIINFEKL epitope was inserted in-frame at the 3′ end of the env gene (B) Detailed strategy for the generation of F-MuLV-OVA. Oligonucleotide primers harboring the OVA epitope sequence and hybridizing with the F-MuLV genome at the end of the env gene were used for PCR-based mutagenesis with the permutated molecular clone of F-MuLV as the template. F-MuLV genome sequence and base numbers shown are according to the database information (Z11128). The vertical arrow indicates the site of cleavage that generates fusogenic TM protein and R peptide [60]. (C) Splenocytes from naïve B6AF1 mice were infected in vitro with either F-MuLV or F-MuLV-OVA. Cells were then cocultured with CD8+ T cells purified from (OT-1-Thy1.1× A/WySnJ)F1 mice (OT-1 cell). Shown are representative histograms for CD69 expression on OT-1 cells. (DOC) [file ppat.1003937.s005.doc]

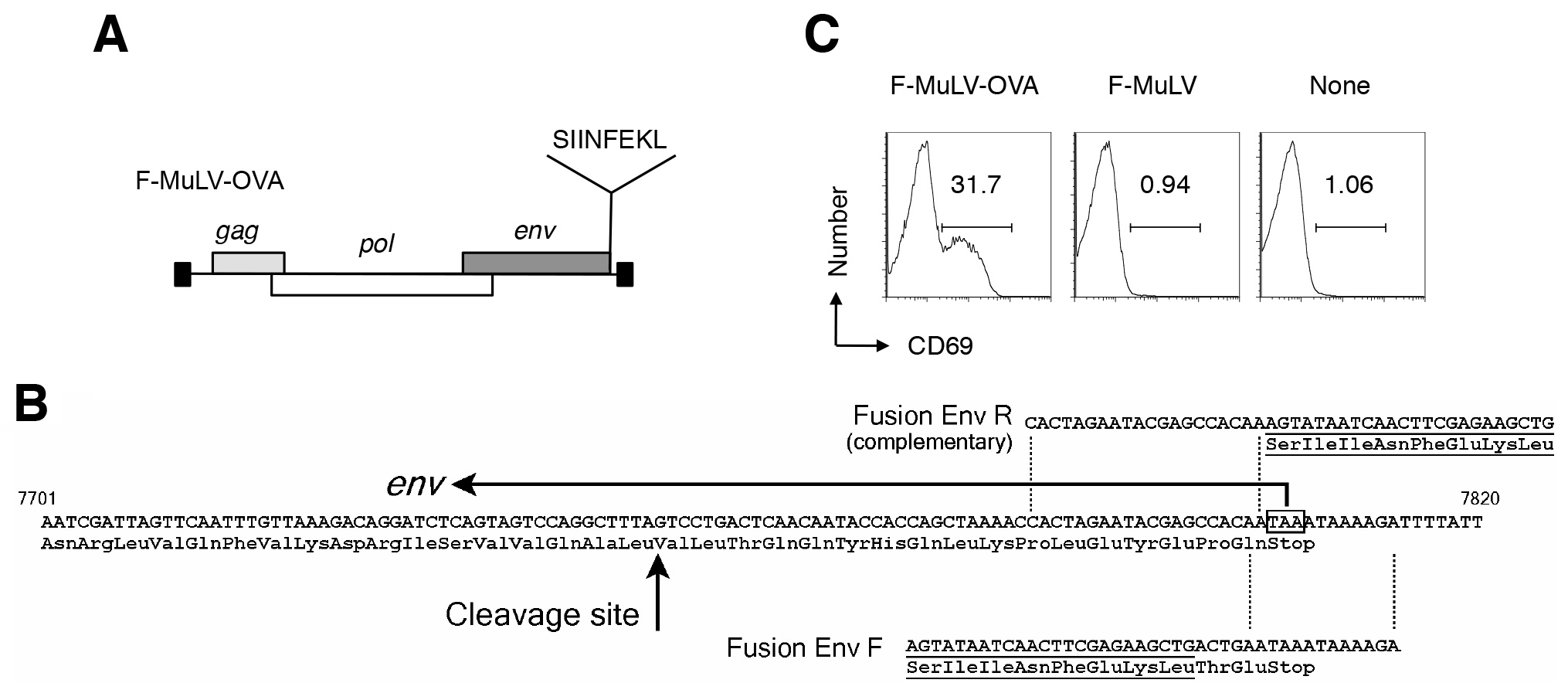


**Figure S5. Construction of F-MuLV-OVA.** (A) Schematic representation of the F-MuLV-OVA construct. A synthetic oligonucleotide encoding the SIINFEKL epitope was inserted in-frame at the 3’ end of the *env* gene (B) Detailed strategy for the generation of F-MuLV-OVA. Oligonucleotide primers harboring the OVA epitope sequence and hybridizing with the F-MuLV genome at the end of the *env* gene were used for PCR-based mutagenesis with the permutated molecular clone of F-MuLV as the template. F-MuLV genome sequence and base numbers shown are according to the database information (Z11128). The vertical arrow indicates the site of cleavage that generates fusogenic TM protein and R peptide . (C) Splenocytes from naïve B6AF_1_ mice were infected in vitro with either F-MuLV or F-MuLV-OVA. Cells were then cocultured with CD8^+^ T cells purified from (OT-1-Thy1.1 × A/WySnJ)F_1_ mice (OT-1 cell). Shown are representative histograms for CD69 expression on OT-1 cells.
