# Supplementary material for: Infection of Adult Thymus with Murine Retrovirus Induces Virus-Specific Central Tolerance That Prevents Functional Memory CD8+ T Cell Differentiation
Source: PLoS Pathog. 2014 Mar 20;10(3):e1003937. doi: 10.1371/journal.ppat.1003937 (PMC3961338; doi:10.1371/journal.ppat.1003937)

**Figure S6. FACS profiles of cells from FTOC.** Experiments were performed as described for Figure 6. Either tumor cells (A) or thymic cell populations purified from FV-OVA-infected mice (B) were used as the third population. Shown are representative dot plots of positive control settings (A) and experimental settings (B).


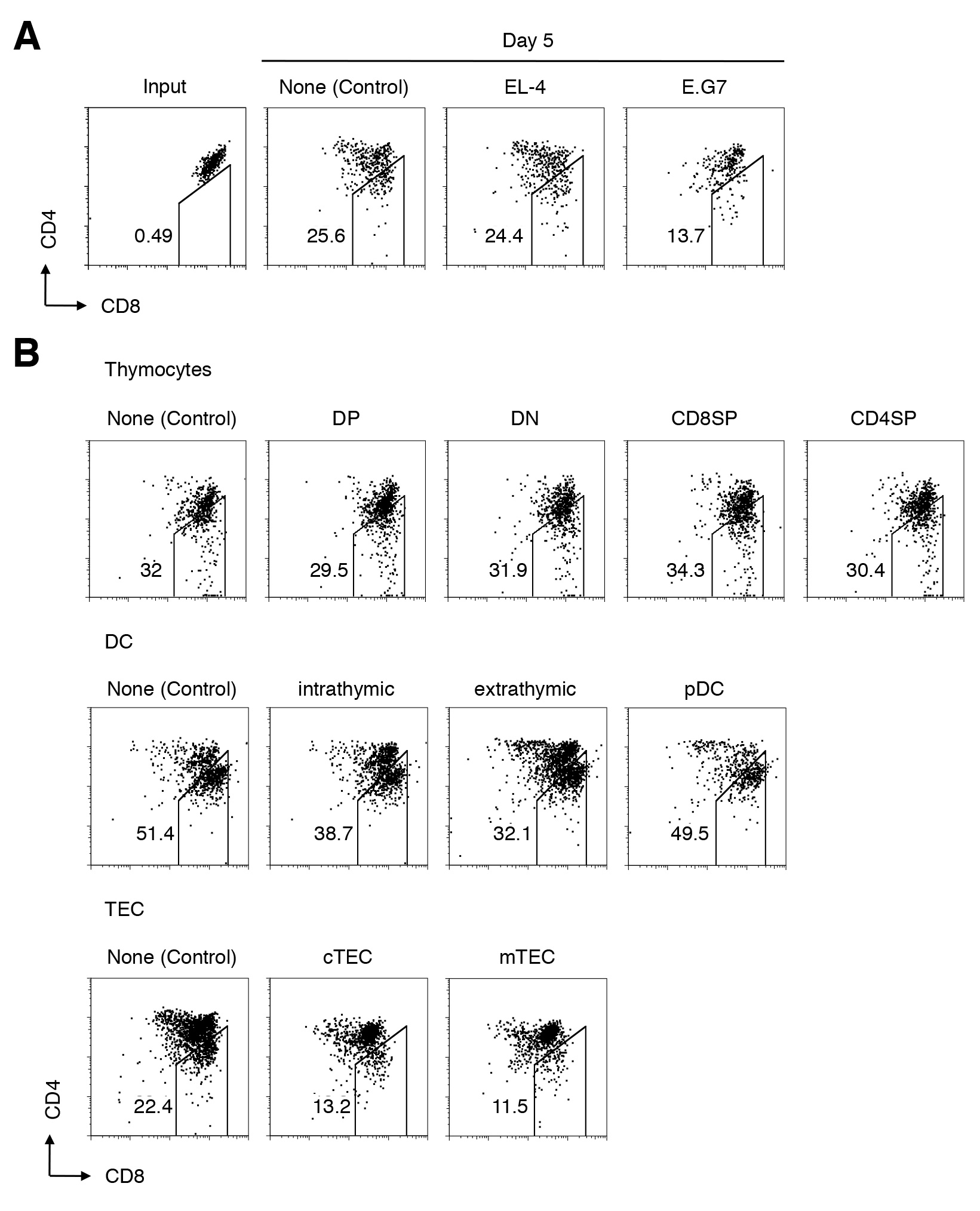

Supplement: Figure S6 — FACS profiles of cells from FTOC. Experiments were performed as described for Figure 6. Either tumor cells (A) or thymic cell populations purified from FV-OVA-infected mice (B) were used as the third population. Shown are representative dot plots of positive control settings (A) and experimental settings (B). (DOC) [file ppat.1003937.s006.doc]
