# Supplementary material for: Infection of Adult Thymus with Murine Retrovirus Induces Virus-Specific Central Tolerance That Prevents Functional Memory CD8+ T Cell Differentiation
Source: PLoS Pathog. 2014 Mar 20;10(3):e1003937. doi: 10.1371/journal.ppat.1003937 (PMC3961338; doi:10.1371/journal.ppat.1003937)
Supplement: Figure S7 — Post-thymic maturation of CD8+ RTEs in mice chronically infected with FV. (Rag1-GFP×A.WySnJ)F1 mice were infected with 1,000 SFFU of FV. Splenocytes were isolated at the indicated time-points and stained with the indicated Abs. Shown are dot plots for GFP expression among CD4+ and CD8+ T cells at day 42 after infection (A), and actual numbers of GFP+CD8+ T cells in either FV-infected or age-matched naïve animals (B). No significant difference was observed between the groups. (C–D) Shown are representative staining patterns for PD-1, CD69 and CD44 of GFP+CD8+ T cells or GFP−CD8+ T cells (C), and for CD24, Qa2 and CD127 of GFP−, GFPlo or GFPhi cells (D). (E–F) Splenocytes were stimulated in vitro with anti-CD3 Ab. The intracellular expression of IFN-γ and IL-2 were then measured by flow cytometry. Shown are representative staining patterns for IFN-γ and CD107a of GFP+CD8+ T cells (E), and frequencies of IFN-γ+ cells and IL-2+ cells among GFP+CD8+ T cells (F). Each symbol represents an individual mouse. Average percentages were compared between uninfected and FV-infected groups by two-way ANOVA with Bonferroni's corrections for multiple comparisons, and no significant difference was detected. Data are representative of two independent experiments with essentially equivalent results. (DOC) [file ppat.1003937.s007.doc]

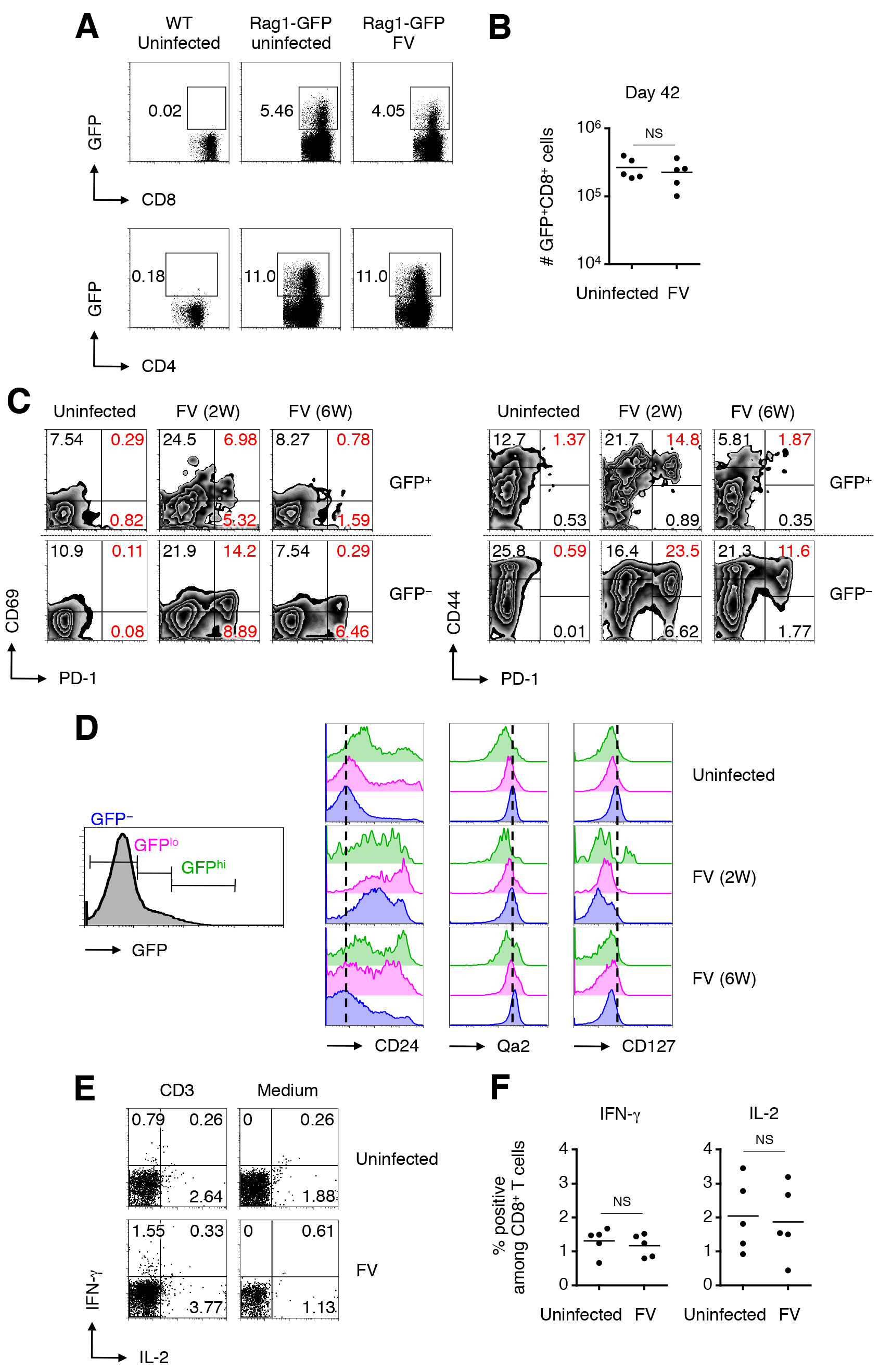
**Figure S7. Post-thymic maturation of CD8^+^ RTEs in mice chronically infected with FV.** (*Rag1*-GFP × A.WySnJ)F_1_ mice were infected with 1,000 SFFU of FV. Splenocytes were isolated at the indicated time-points and stained with the indicated Abs. Shown are dot plots for GFP expression among CD4^+^ and CD8^+^ T cells at day 42 after infection (A), and actual numbers of GFP^+^CD8^+^ T cells in either FV-infected or age-matched naïve animals (B). No significant difference was observed between the groups. (C-D) Shown are representative staining patterns for PD-1, CD69 and CD44 of GFP^+^CD8^+^ T cells or GFP^−^CD8^+^ T cells (C), and for CD24, Qa2 and CD127 of GFP^−^, GFP^lo^ or GFP^hi^ cells (D). (E-F) Splenocytes were stimulated *in vitro* with anti-CD3 Ab. The intracellular expression of IFN-γ and IL-2 were then measured by flow cytometry. Shown are representative staining patterns for IFN-γ and CD107a of GFP^+^CD8^+^ T cells (E), and frequencies of IFN-γ^+^ cells and IL-2^+^ cells among GFP^+^CD8^+^ T cells (F). Each symbol represents an individual mouse. Average percentages were compared between uninfected and FV-infected groups by two-way ANOVA with Bonferroni’s corrections for multiple comparisons, and no significant difference was detected. Data are representative of two independent experiments with essentially equivalent results.
